# The skin microbiome stratifies patients with cutaneous T cell lymphoma and determines event-free survival

**DOI:** 10.1038/s41522-024-00542-4

**Published:** 2024-08-29

**Authors:** Philipp Licht, Nazzareno Dominelli, Johannes Kleemann, Stefan Pastore, Elena-Sophia Müller, Maximilian Haist, Kim Sophie Hartmann, Henner Stege, Matthias Bros, Markus Meissner, Stephan Grabbe, Ralf Heermann, Volker Mailänder

**Affiliations:** 1 University Medical Centre Mainz, Department of Dermatology, Mainz, Germany; 2 Johannes Gutenberg-University, Institute of Molecular Physiology (imP), Biocenter II, Microbiology and Biotechnology, Mainz, Germany; 3 University Hospital Frankfurt, Department of Dermatology, Venerology and Allergology, Frankfurt am Main, Germany; 4 University Medical Centre Mainz, Institute of Human Genetics, Mainz, Germany; 5 Johannes Gutenberg-University, Institute of Pharmaceutical and Biomedical Sciences, Mainz, Germany; 6 Max Planck Institute for Polymer Research, Mainz, Germany

**Keywords:** Clinical microbiology, Metagenomics, Microbiome, Pathogens

## Abstract

Mycosis fungoides (MF) is the most common entity of Cutaneous T cell lymphomas (CTCL) and is characterized by the presence of clonal malignant T cells in the skin. The role of the skin microbiome for MF development and progression are currently poorly understood. Using shotgun metagenomic profiling, real-time qPCR, and T cell receptor sequencing, we compared lesional and nonlesional skin of 20 MF patients with early and advanced MF. Additionally, we isolated *Staphylococcus aureus* and other bacteria from MF skin for functional profiling and to study the *S. aureus* virulence factor *spa*. We identified a subgroup of MF patients with substantial dysbiosis on MF lesions and concomitant outgrowth of *S. aureus* on plaque-staged lesions, while the other MF patients had a balanced microbiome on lesional skin. Dysbiosis and *S. aureus* outgrowth were accompanied by ectopic levels of cutaneous antimicrobial peptides (AMPs), including adaptation of the plaque-derived *S. aureus* strain. Furthermore, the plaque-derived *S. aureus* strain showed a reduced susceptibility towards antibiotics and an upregulation of the virulence factor *spa*, which may activate the NF-κB pathway. Remarkably, patients with dysbiosis on MF lesions had a restricted T cell receptor repertoire and significantly lower event-free survival. Our study highlights the potential for microbiome-modulating treatments targeting *S. aureus* to prevent MF progression.

## Introduction

Mycosis fungoides (MF) is a lymphoproliferative disorder of skin homing T cells and the most common entity of the heterogenous group of cutaneous T cell lymphoma (CTCL)^1^. MF patients usually present with multiple lesional areas in the skin that are classified into patches, plaques, and tumours, based on the extent of neoplastic T cell infiltration, the degree of inflammation and disease activity^1,2^. Clinical stages range from IA to IVB and consider, besides the skin lesions, also involvement of extracutaneous sites like the blood compartment^3^. Most patients keep in early-stages for their whole life (IA-IIA), yet up to one third of patients progresses to advanced stages within 10 years after diagnosis. In such cases, the 5-year overall survival drops dramatically from ~80% in early stages to 18% in most advanced stages^4,5^ and may be accompanied by dissemination of malignant T cells into other organs^1,2^. Despite a better understanding of CTCL in recent decades, genomic drivers of MF pathogenesis remain elusive^1,2,6–9^. In consequence, there are no effective or sustainable therapies for advanced stage CTCL with the exception of hematopoietic stem cell transplantation, which comes with severe side effects^10^. As cure is not achievable but long-term survival is possible, maintenance of stable disease is aspired^11,12^.

Postulated mechanisms of MF pathogenesis include the persistence of viral or bacterial agents from the skin microbiome that maintain chronic T cell expansion and cutaneous inflammation^13,14^. The importance of the skin microbiome for MF pathogenesis has previously been shown in a CTCL mouse model. Here, CTCL progression was attenuated under germ-free conditions and aggravated in the presence of microbiota^15^. In addition, systemic antibiotic therapy of MF patients sometimes leads to remission^16–18^, and a number of studies suggest that *Staphylococcus aureus* can facilitate MF progression^19,20^. However, *S. aureus* is also a commensal of the physiological skin flora in healthy individuals^21^ and not all MF patients benefit from *S. aureus* eradication^18,22^. Therefore, we hypothesized that MF patients have a more significant disturbance of the skin microbiome beyond the mere presence of pathogenic microbes like *S. aureus*.

So far, several reports characterized the MF skin microbiome^23–27^ and indicated that (a) a destabilized microbiome and^23,28^ (b) the abundance of certain microbial genera like *Cutibacteria* or *Staphylococci*^24,25^ might be associated with disease severity. However, statistical significance was largely not reached, likely because of small sample sizes^24^, characterization approaches that lacked resolution at the microbial species level, or because samples were not categorized based on clinical or lesional stages^28^.

In this study, we investigated the role of the skin microbiome for MF pathogenesis in 20 patients with early- and advanced-stage MF using metagenomic sequencing, RT-qPCR and T cell receptor sequencing (TCRseq). We show that a subgroup of patients exhibited a substantial dysbiosis on MF lesions with concomitant outgrowth of *S. aureus* on plaque (termed ΔSA-positive) as compared to nonlesional skin, while the other subgroup had a balanced microbiome on these lesions (termed ΔSA-neutral). The perturbations in the former group might be caused by ectopic antimicrobial peptides eradicating the normal skin flora. While in the ΔSA-neutral subgroup physiological microbes with anti-*S. aureus* activity accumulated, the ΔSA-positive subgroup was dominated by *S. aureus*. Clinical isolate of this species showed increased adaptation to AMPs, which likely contributed to its outgrowth. Furthermore, *S. aureus* strains from lesional skin showed resistance towards common antibiotics and were highly virulent, thereby evading the host immune response and assaulting the T cell receptor repertoire. In addition, we observed a potential gain-of-function mutation in the virulence factor *spa* possibly rendering it highly potent to activate the NF-κB axis, a frequently overactivated feature in MF patient subgroups with progressive disease^29–31^. In accordance, we observed considerably reduced event-free survival in the ΔSA-positive subgroup. Our study highlights the importance of the skin microbiome for MF pathogenesis, thus opening new options for the treatment of MF.

## Results

### Characteristics of patient cohort and clinical specimens

To investigate the microbiome on MF lesions, we analysed metagenomic samples from patches (n = 19), plaques (n = 15) and nonlesional skin (n = 31) of 20 MF patients (8 females, 12 males) recruited from two skin cancer centres in Germany (Mainz, n = 14 patients and Frankfurt, n = 6 patients). Nonlesional skin from the contralateral body site of the same patients served as controls. Clinical samples were collected from patients with MF stages IA – IIB, with 15 patients displaying early-stage MF (stages IA–IIA), and 5 patients suffering from advanced-stage MF (IIB). We did not include tumour-stage lesions because these often ulcerate and might thereby offer a unique microbial habitat due to their moist wound characteristic^1,32^. The mean age at sampling was 66.4 ± 11.7 years, and the mean age at first diagnosis of MF was 60 ± 11.2 years. Patients were treated with common therapy (Table 1). In addition to metagenomic samples, that were collected for the entire patient cohort, we obtained skin punch biopsies from lesional and adjacent skin, as well as peripheral blood in a subset of patients. RT-qPCR (N = 24) was performed from the skin punch biopsies to study expression levels of antimicrobial peptides (AMPs). T cell receptor sequencing (TCRseq) was carried out on a total of 16 skin samples and 5 blood samples, obtained from 10 patients. Living bacterial isolates were picked from plaque and nonlesional skin of one patient (Pat1) and from healthy subjects. The patients clinical course including events (defined as death, start of new therapy and disease progression) and the time from observation start to event (TTE) were assessed. A summary of the patient characteristics is given in Table 1.

**Table 1:** Patient characteristics, therapy regimen and clinical outcomes of the study cohort

Patient ID	Study Site	Sex	Age at Sampling (years)	Clinical Stage at Sampling	Metagenomics:Stage of Lesions (Body Site)	RT-qPCR: Stage of Lesions (Body Site)	TCRseq:Tissues included	ΔSA- subgroup	Therapy	Event	TTE (months)	Follow-up Time (months)
Pat1	MZ	f	81	IA	Plaque (hip), Patch (forearm)	Plaque (hip)	Skin (Plaque), Blood	positive	PUVA, Mechlorethamine	Yes	2.60	17.56
Pat2	MZ	f	57	IB	Plaque (thigh), Plaque (abdomen)	NA	NA	neutral	PUVA, UVB, topical steroids, Pimecrolimus, MTX, Bexarotene, Mogamulizumab	Yes	18.41	18.41
Pat3	MZ	f	47	IB	Patch (thigh)	Patch (thigh)	NA	neutral	topical steroids	Censored	Censored	17.95
Pat4	MZ	m	70	IIB	Plaque (upper back),Plaque (flank)	Plaque (upper back)	Skin (Plaque and nonlesional), Blood	positive	topical steroids, Bexarotene, Mogamulizumab, RTx	Yes	0.92	9.99
Pat5	MZ	f	73	IB	Patch (thigh),Patch (forearm)	Patch (thigh)	Skin (Patch and nonlesional)	neutral	topical steroids	Yes	13.87	17.10
Pat6	MZ	f	81	IA	Patch (breast)	Patch (breast)	Skin (Patch and nonlesional)	neutral	topical steroids, Mechlorethamine	Censored	Censored	15.55
Pat7	MZ	m	54	IB	Patch (forearm), Plaque (flank)	Plaque (flank)	Skin (Plaque and nonlesional)	neutral	PUVA, PEG-IFNα	Censored	Censored	16.11
Pat8	MZ	m	82	IIB	Plaque (head, parietal), Patch (upper back)	Patch (upper back)	Skin (Patch), Blood	positive	topical steroids, MTX, RTx	Yes	2.53	2.53
Pat9	MZ	m	63	IB	Plaque (forearm)	Plaque (forearm)	Skin (Plaque)	neutral	Topical steroids, PUVA, RTx	Yes	6.41	14.27
Pat10	MZ	m	49	IB	Patch (gluteus right), Patch (gluteus left)	Patch (gluteus left)	Skin (Patch)	neutral	NA	LFU	LFU	LFU
Pat11	MZ	m	61	IIB	Patch (wrist)	NA	NA	neutral	NA	LFU	LFU	LFU
Pat12	MZ	m	88	IA	Patch (abdomen), Patch (thigh)	Patch (thigh)	Skin (Patch and nonlesional), Blood	neutral	no special therapy, only moisturizing cremes	Censored	Censored	11.51
Pat13	MZ	m	66	IB	Patch (lower leg, front), Patch (lower leg, back)	Patch (lower leg, back)	NA	neutral	topical steroids	Censored	Censored	11.51
Pat14	MZ	m	62	IB	Plaque (forearm), Plaque (lower leg, front)	Plaque (lower leg, front)	Skin (Plaque and nonlesional), Blood	neutral	topical steroids, Silver iodide	Censored	Censored	11.05
Pat15	FFM	f	73	IB	2x Plaque (lower leg, front and gluteus)	NA	NA	positive	MTX, RTx	Yes	2.83	11.57
Pat16	FFM	f	58	IA	Plaque (scapula)	NA	NA	positive	MTX, Bexarotene	Yes	4.87	11.51
Pat17	FFM	m	83	IIB	2x Patch (hip and rips)	NA	NA	neutral	Bexarotene	Yes	7.00	7.00
Pat18	FFM	f	61	IB	2x Patch (thigh dorsal & abdominal)	NA	NA	neutral	no special therapy	Censored	Censored	9.99
Pat19	FFM	m	54	IIB	Patch (shoulder), Plaque (thigh)	NA	NA	neutral	Bexarotene	Censored	Censored	9.67
Pat20	FFM	m	65	IB	Plaque (forearm)	NA	NA	neutral	Bexarotene	Censored	Censored	11.11

*f* female, m male, *MZ* Mainz, Germany; *FFM* Frankfurt am Main, Germany; *PUVA* psoralen and UVA, *MTX* methotrexate, *PEG-IFNα* pegylated interferon alpha, *RTx* radiation therapy, *NA* not available, *LFU* lost to follow-up, *TTE* Time To Event.

### Analysis of rarefied microbial reads shows a dysbiosis on lesional MF skin

**Fig. 1:**
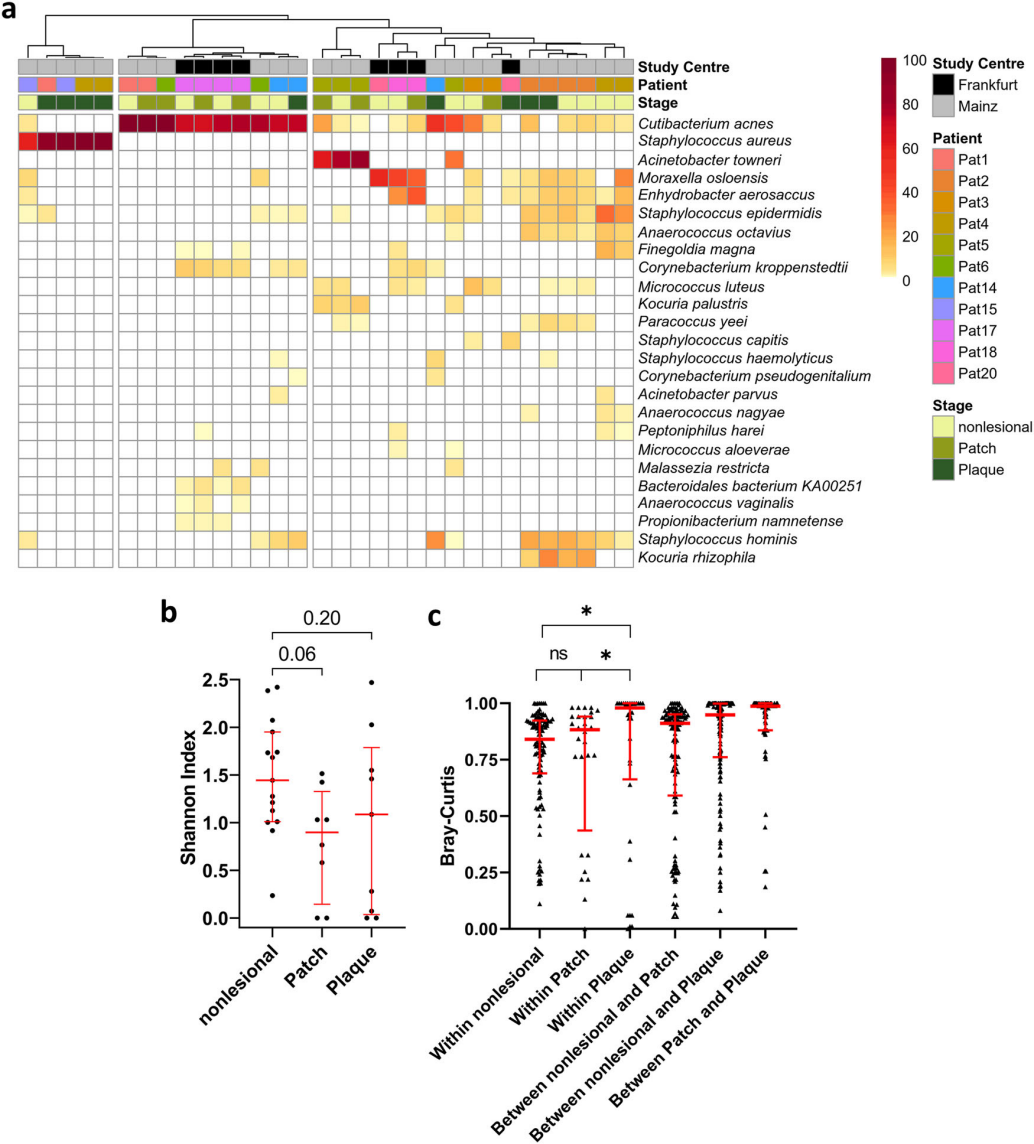
Analysis of rarefied metagenomic profiles. **a** Clustered taxonomic profiles of the 25 most abundant species present on nonlesional skin, patch, and plaque. Reads were rarefied to common depth prior to assignment of taxonomy. *n* = 32 metagenomic samples were left after rarefaction and included in the clustered heatmap. Displayed is the relative abundance in percent. **b** α-diversity (Shannon-Index) of *n* = 32 rarefied metagenomic samples (15 nonlesional, 8 patch, 9 plaque), one-way ANOVA corrected for multiple comparisons (Dunnett’s test), dot plots with median and interquartile range. **c** β-diversity (Bray-Curtis) dissimilarities, pairwise PERMANOVA, dot plots with median and interquartile range. 2 degrees of freedom in **b** and **c**.

In a first analysis we clustered taxonomic profiles that were generated from rarefied metagenomic samples (Fig. 1a). As expected, clustering showed a strong grouping of specimens derived from the same patient, which justified our approach of using intra-patient controls^21^. By contrast, we observed no significant differences in the microbiome composition between the two study centres, and therefore did not consider these in subsequent analysis (see also Supplementary Fig. 1d). Overall, lesional samples appeared to have a lower microbial diversity compared to nonlesional controls. Remarkably, some patch and plaque samples from different patients clustered together, showing that the microbiome on lesional skin was altered in a uniform manner. To test whether the composition of the microbial community on patches and plaques differed from that of nonlesional skin, we calculated Shannon indices and Bray-Curtis dissimilarities from rarefied microbial reads. The α-diversity was reduced on MF lesions, with patch stage showing the strongest effect, but significance was not reached (Fig. 1b). However, β-diversity revealed a significant instability of the microbiome on MF lesions that increased with exacerbation of MF lesions (Fig. 1c). Moreover, α- and β-diversity of plaque skin exhibited a bimodal distribution. Several samples showed a strongly decreased Shannon Index and an affected microbiome stability, whereas others resembled the diversity-metrics of nonlesional skin. Notably, except for age, no significant associations with other demographics of the study cohort were found (Supplementary Fig. 1). We found a slightly, rather heterogenous decrease in microbial diversity with increasing age. Decreased microbial diversity is a known feature in elderly people^33^ and was therefore to be expected. Interestingly, elder people were described to have reduced colonization with *S. aureus*^34^. Together, these results showed that dysbiosis is a common feature of MF lesions, which increased with exacerbation.

### *S. aureus* colonization stratifies the MF patient cohort into two subgroups with distinct microbiome patterns

**Fig. 2:**
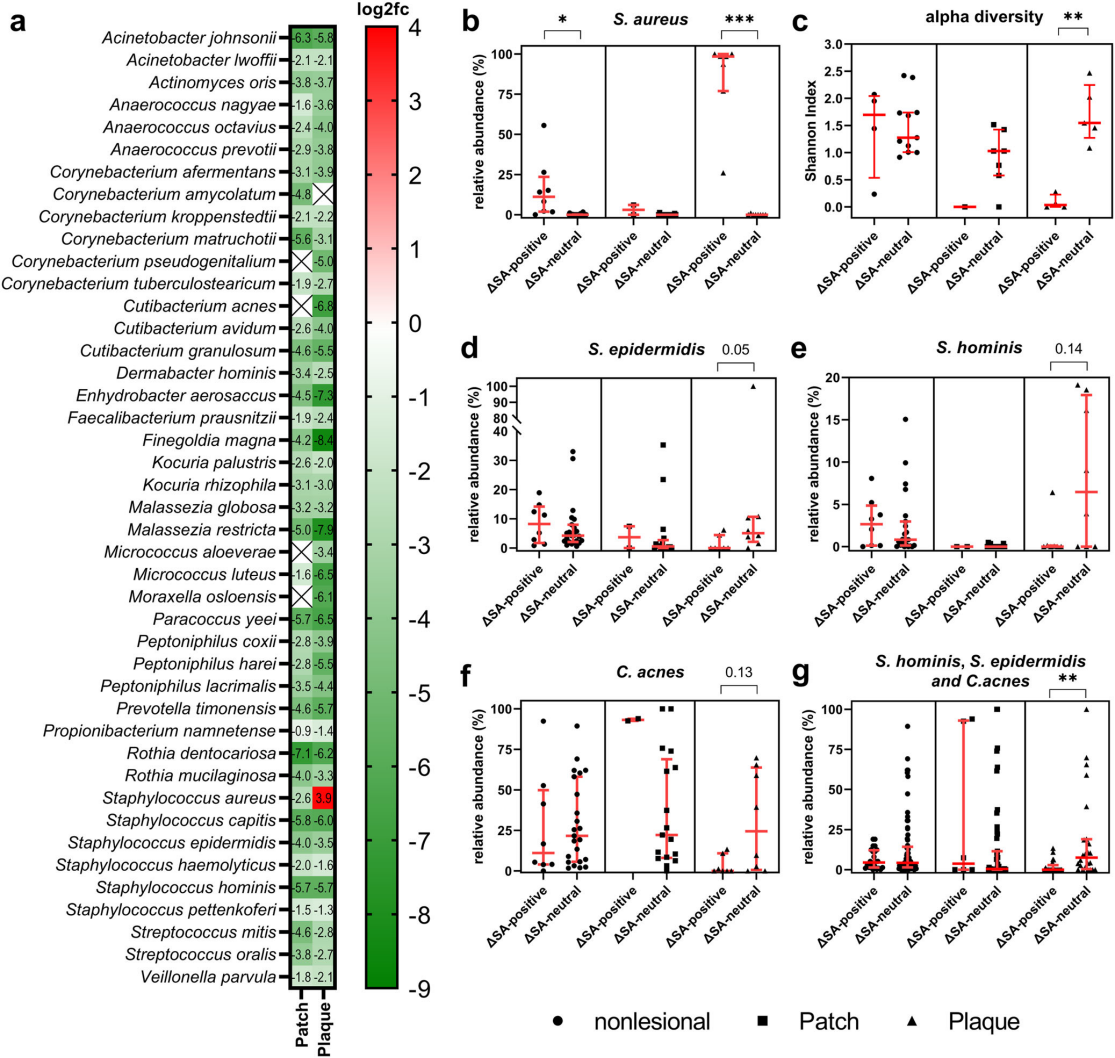
Differential abundance analysis of microbial species for N = 65 metagenomic samples. **a** log2 fold-change (log2fc) of microbial species present on patch (n = 19) and plaque (n = 15) compared to nonlesonial skin (n = 31). **b** *S. aureus* relative abundance increases from nonlesional skin to plaque in the ΔSA-positive subgroup, while this is not the case for the other subgroup (ΔSA-neutral). **c**–**g**, Shannon-Index and relative abundances of bacteria stratified to ΔSA-subgroups. Shannon Index is based on n = 32 metagenomic samples rarefied to common depth as in Fig. 1b. Depicted are dot plots with median and interquartile range, Kruskal-Wallis test (**b**, **d**–**g**) or one-way ANOVA with correction for multiple comparisons.

To understand which species accounted for the observed microbiome patterns on lesional skin, we set up a generalized linear mixed model (GLMM) using MaAsLin2^35^. We tested for differences in the abundance of microbial species on patches and plaques compared to nonlesional skin while adjusting for sequencing depth and the individuality of the patient’s microbiome. Using this approach, we found that *S. aureus* was highly enriched on plaque while all other significantly associated microbial species were extremely reduced on patch and plaque (Fig. 2a, Supplementary Table 3). Among them were *S. hominis*, *S. epidermidis* and *Cutibacterium acnes*. These commensals can control *S. aureus* growth in other inflammatory skin conditions^36–39^ and confer decreased release of inflammatory cytokines as well as recruitment of leucocytes in skin wounding healing^40^.

When plotting the relative abundance of *S. aureus* patient-wise and differentiating between lesional stages, we noted a patient stratification into two subgroups: In one subgroup, the relative abundance of *S. aureus* did not change from nonlesional skin to plaque stage. In the other subgroup however *S. aureus* abundance substantially increased from nonlesional skin to plaque stage. We therefore referred to the first group as Δ *S. aureus*-neutral (ΔSA-neutral) and to the other group as Δ *S. aureus*-positive (ΔSA-positive) (Fig. 2b). Strikingly, when stratifying the α-diversity (Shannon Index) to the defined patient subgroups, the observed bimodality resolved: ΔSA-positive patients exhibited a significantly reduced α-diversity on plaques, and ΔSA-neutral patients in turn had a plaque microbiome which was as diverse as that on nonlesional skin (Fig. 2c). Likewise, the three commensals with anti-*S. aureus* activity were more prominent on plaques of ΔSA-neutral patients, albeit this association was above statistical significance (*p* = 0.05–0.14). This might be attributed to the fact that every plaque lesion of ΔSA-neutral patients was dominated by only one of the three commensals with anti-*S. aureus* action while the other two were minor constituents of the skin flora (Fig. 2d–f). When compiling *S. hominis*, *S. epidermidis* and *C. acnes*, *S. aureus*-inhibiting microbes were significantly more abundant on plaque than on nonlesional skin of ΔSA-neutral patients (Fig. 2g).

### Increased and sustained expression of cutaneous antimicrobial peptides could lead to skin dysbiosis

**Fig. 3:**
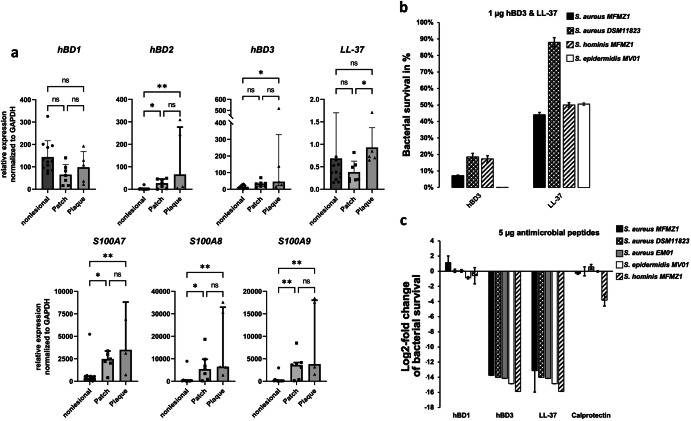
Cutaneous AMPs disturb the skin microbiome. **a** RNA expression level of antimicrobial peptides. Punch biopsies were taken from MF lesions (n = 7 patch, n = 5 plaque) and adjacent nonlesional skin (n = 12). Expression levels were determined with RT-qPCR and normalized to GAPDH. S100A8 and S100A9 together form the heterodimer calprotectin. Depicted are bar plots with median and interquartile range, Kruskal-Wallis test with multiple comparison correction. **b**, **c** Activities of AMPs against Staphylococci isolates. Survival of clinical isolates *S. aureus* MFMZ1 and *S. hominis* MFMZ1, clinical control strain *S. aureus* DSM11823 as well as isolates from healthy subjects *S. aureus* EM01 and *S. epidermidis* MV01 in presence of **b**, 1 µg and **c**, 5 µg AMPs. Bacterial survival was assessed by comparing number of colony forming units (CFU) with and without AMP treatment for each isolate and AMP. Displayed are bacterial survival for three biological replicates in percent (**b**) and log2-fold change (**c**), respectively.

The skin expresses a diverse repertoire of antimicrobial peptides (AMPs) to control microbial colonization and ensure epithelial integrity^41,42^. Under steady-state conditions, AMPs are constitutively produced at low rates but increase upon injury or inflammation^41,42^. MF is characterized by an inflammatory microenvironment and lesions might persist over long time periods^43^. In order to evaluate whether cutaneous AMPs may have contributed to the skin dysbiosis we obtained skin punch biopsies from the same MF lesions sampled for metagenomic profiling and analysed AMP expressions levels using RT-qPCR. Adjacent nonlesional skin served as control. RNA expression levels of the AMPs *hBD2*, *hBD3*, *S100A7*, and the calprotectin forming *S100A8* and *S100A9* were significantly increased in MF lesions but did not significantly differ between patch and plaque stage (Fig. 3a). This observation is consistent with the fact that these AMPs are known to be regulated by microbial recognition mechanisms^42,44^. In contrast, *hBD1* and *LL-37* are known to be constitutively expressed rather than inducible^42,44^, which is in agreement with our results (Fig. 3a).

Next, we examined whether ectopic AMP levels affect growth of cutaneous bacterial species found on MF lesions. Clinical isolates of *S. aureus* and S*. hominis* were picked from plaque skin of Pat1 (termed *S. aureus* MFMZ1 and *S. hominis* MFMZ1, respectively). *S. aureu*s (EM01) and *S. epidermidis* (MV01) picked from 2 healthy subjects served as negative controls. Another clinical *S. aureus* isolate historically picked from pleural fluid back in 1884 (*S. aur*eus Rosenbach 1884, strain DSM11823) served as positive control^45^. All bacterial samples were exposed to increasing concentrations of hBD1, hBD3, LL-37 and calprotectin (a heterodimer consisting of S100A8 and S100A9).

Clinical isolates *S. aureus* MFMZ1, *S. hominis* MFMZ1 and *S. aureus* DSM 11823 survived at low concentrations of hBD3 (1 µg), while healthy subject-derived *S. epidermidis* MV01 was eradicated. All strains survived at low concentrations of LL-37 (1 µg, Fig. 3b). This was to be expected, because LL-37 was constitutively expressed in nonlesional skin and was also not induced by the disease (Fig. 3a). Hence, bacterial skin colonization requires resistance to LL-37 at least at low concentrations. Interestingly, *S. aureus* MFMZ1 showed the least reduction in survival at high concentrations of hBD3 and LL-37 compared to all other strains tested. (Fig. 3c). Furthermore, exposure to calprotectin resulted in decreased survival of only *S. hominis* MFMZ1, a bacterium with potential anti-*S. aureus* properties^36–40^. Surprisingly, hBD1 had a paradoxical effect on *S. aureus* MFMZ1, even augmenting its survival, while the survival of all other tested strains remained unchanged or decreased (Fig. 3c).

These data demonstrate that clinical isolates of *S. aureus*, and particularly that obtained from plaque of an MF patient, have considerable survival advantages under ectopic AMP application. As neoplastic T cells and reactive leucocyte infiltrate accumulate in MF lesions over time, plaques can be expected to persist longer than patches and, accordingly, the microbiome on plaques is exposed longer to high AMP levels than the microbiome on patches (and nonlesional skin). Given the fact that skin dysbiosis is most accentuated in patch stage (Fig. 2a), our results collectively indicate that most of the physiological skin flora is eradicated upon onset of increased AMP production. With ongoing persistence of MF lesions and high AMP levels, MF skin commensals eventually adopt to the new environmental conditions and (re-) colonize MF lesions.

### MF skin lesions are colonized by distinct *S. aureus* strains that outgrow other MF skin commensals

**Fig. 4:**
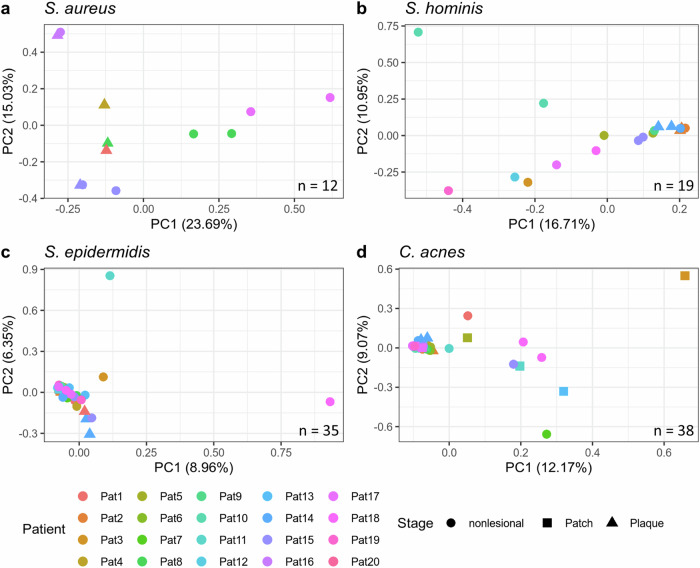
PCA of the gene repertoire in strains of four different microbial species present in the skin microbiome of MF patients. Only metagenomic samples with sufficient coverage of the species of interest are shown. S. aureus is the only species that exhibited strain differences between MF lesions and nonlesional skin, with principal component (PC) 1 being the discriminating factor. Pat15 and Pat16 each had the same strain present on plaque and nonlesional skin, indicating that *S. aureus* present on plaque spread into nonlesional areas of the skin (**a**). For *S. hominis* (**b**), *S. epidermidis* (**c**) and *C. acnes* (**d**) no such differences were found.

We next asked why *S. aureus* outgrows only in the ΔSA-positive subgroup. Given the substantial survival advantages of *S. aureus* under high AMP expression levels and the observation of skin dysbiosis exclusively in the ΔSA-positive subgroup (Fig. 2c), we first reasoned that cutaneous AMP levels of the ΔSA-neutral subgroup would be lower compared to the ΔSA-positive subgroup. Surprisingly, the AMP expression levels did not significantly differ between lesions of ΔSA-neutral and ΔSA-positive patients (Supplementary Fig. 2). There must hence be another factor or event allowing *S. aureus* to accumulate and outgrow competitive commensals. A potential explanation might be that *S. aureus* strains colonizing MF lesions differ from their nonlesional counterparts, since *S. aureus* is a common commensal of human skin flora but also a frequent pathogen in many diseases^21,32^. To test this hypothesis, we used the tool PanPhlAn^46^ to profile bacterial strains in the MF microbiome by the presence and absence of genes in the respective species’ pangenomes. Dimensionality reduction via principal component analysis (PCA) revealed that strains of *S. hominis, S. epidermidis* and *C. acnes* did not differ between MF lesions and nonlesional skin. In contrast, *S. aureus* strains present on plaques had a unique gene repertoire, clearly demonstrating that lesional *S. aureus* were of a different strain than their nonlesional counterparts (Fig. 4).

**Fig. 5:**
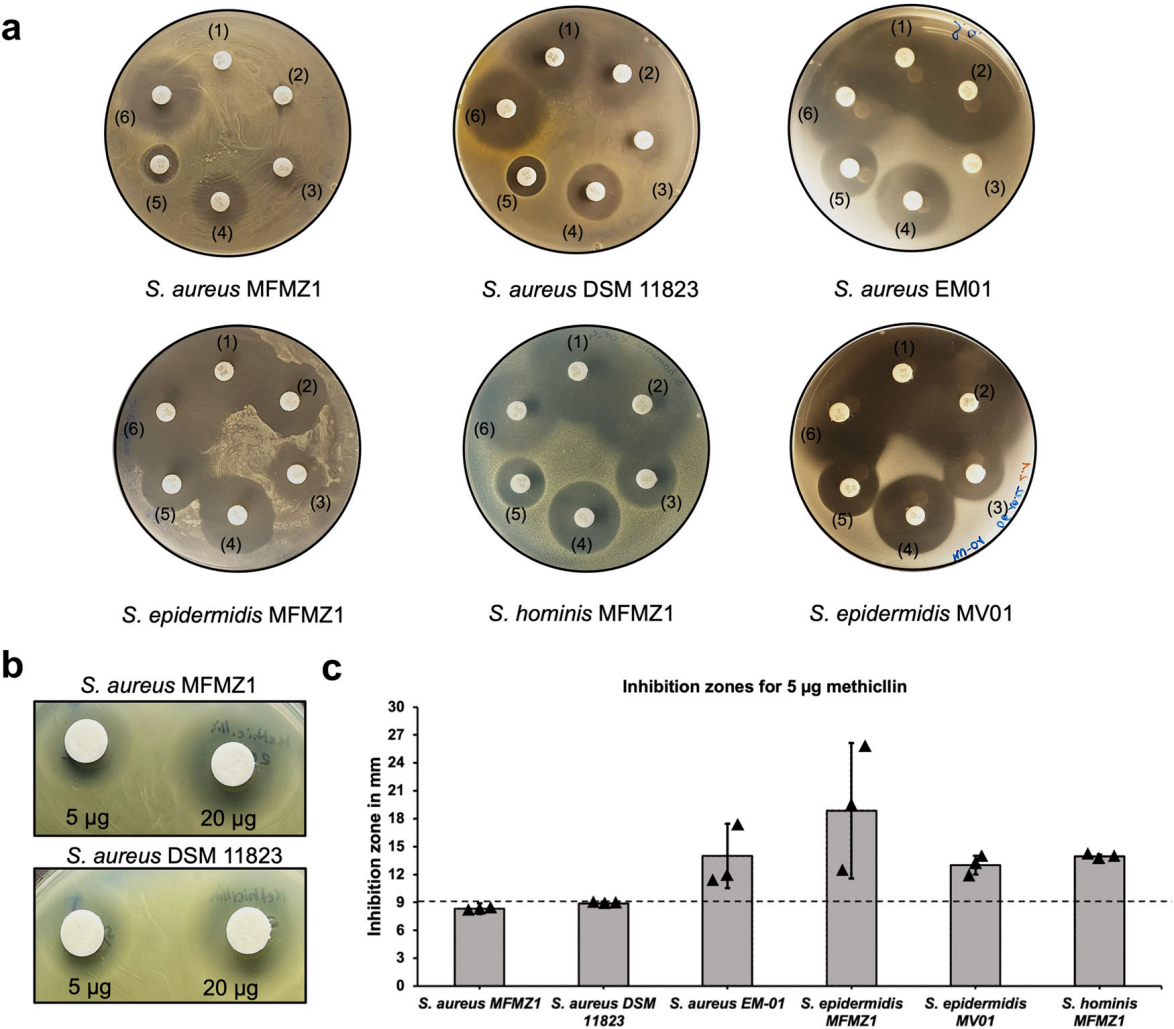
*Antibiotic diffusion assay.* The patient derived isolates *S. aureus* MFMZ1, *S. epidermidis* MFMZ1, and *S. hominis* MFMZ1 as well as positive control *S. aureus* DSM11823 and negative control strains derived from healthy patients *S. aureus* EM01 and *S. epidermidis* MV01 were tested on resistance towards a set of commonly used antibiotics in the clinic. **a** The antibiotics were applied using a Sensi-Disk dispenser with (1) 25 µg ampicillin, (2) 100 µg carbenicillin, (3) (23.75 µg) sulfamethoxazol + (1.25 µg) trimethoprim, (4) 15 µg erythromycin, (5) 10 µg gentamycin, and (6) 5 µg novobiocin. **b**, **c** The strains were additionally tested on methicillin resistance using 5 µg and 20 µg methicillin applied on sterile filter disks. The inhibition zone diameter for *S. aureus* MFMZ1 and DSM11823 strains was ≤ 9 mm, which indicates resistance towards methicillin according to the clinical and laboratory standards institute (CLSI^125^).

In order to validate these observations, we examined whether strain differences seen in the computational analysis could be transferred to the same living isolates tested before. In a disk diffusion assay, *S. aureus* MFMZ1 was considerably more resistant towards antibiotics typically used in the clinic than all other tested strains (Fig. 5). While Pat1-derived *S. epidermidis* MFMZ1 and healthy subject-derived *S. epidermidis* MV01 showed comparable susceptibilities, *S. aureus* MFMZ1 was notably more resistant than its counterpart from healthy skin (*S. aureus* EM01). Remarkably, the other clinical *S. aureus* DSM11823 strain was sensitive towards both β-lactam antibiotics, ampicillin and carbenicillin, as well as gentamycin, whereas *S. aureus* MFMZ1 was not (Fig. 5, Supplementary Table 1). We could further determine that *S. aureus* MFMZ1 was a methicillin resistant *S. aureus* (MRSA) strain, while *S. aureus* EM01 was not (Fig. 5b, c). *S. aureus* MFMZ1 not only demonstrated reduced susceptibility to antibiotics but also exhibited the strongest adaptation to the AMP conditions found on MF skin (Fig. 3b, c). In particular, this strain was not only resistant to hBD1, but its growth was even promoted by the AMP. Given that the other skin bacteria we tested in this assay did not show this effect, and were less resistant to other AMPs, likely provided *S. aureus* with an advantage over other skin commensals on plaques.

Taken together, *S. aureus* present on MF lesions was of a distinct strain that differed from its nonlesional counterpart, probably outcompeting other skin commensals by adapting more effectively to the unique environmental conditions of MF skin.

### *S. aureus* strains on plaque of ΔSA-positive patients are highly virulent

Because *S. aureus* often acts as a pathogen^32^, we next evaluated the virulent properties of the microbiome present on MF lesions. Utilizing ShortBRED^47^, we profiled the whole metagenome sequencing (WMS) reads against the Virulence Factor Database (VFDB)^48^ to detect the presence of virulence factors. Differences in virulence factor abundance between nonlesional skin and MF lesions were evaluated using MaAsLin 2.

**Fig. 6:**
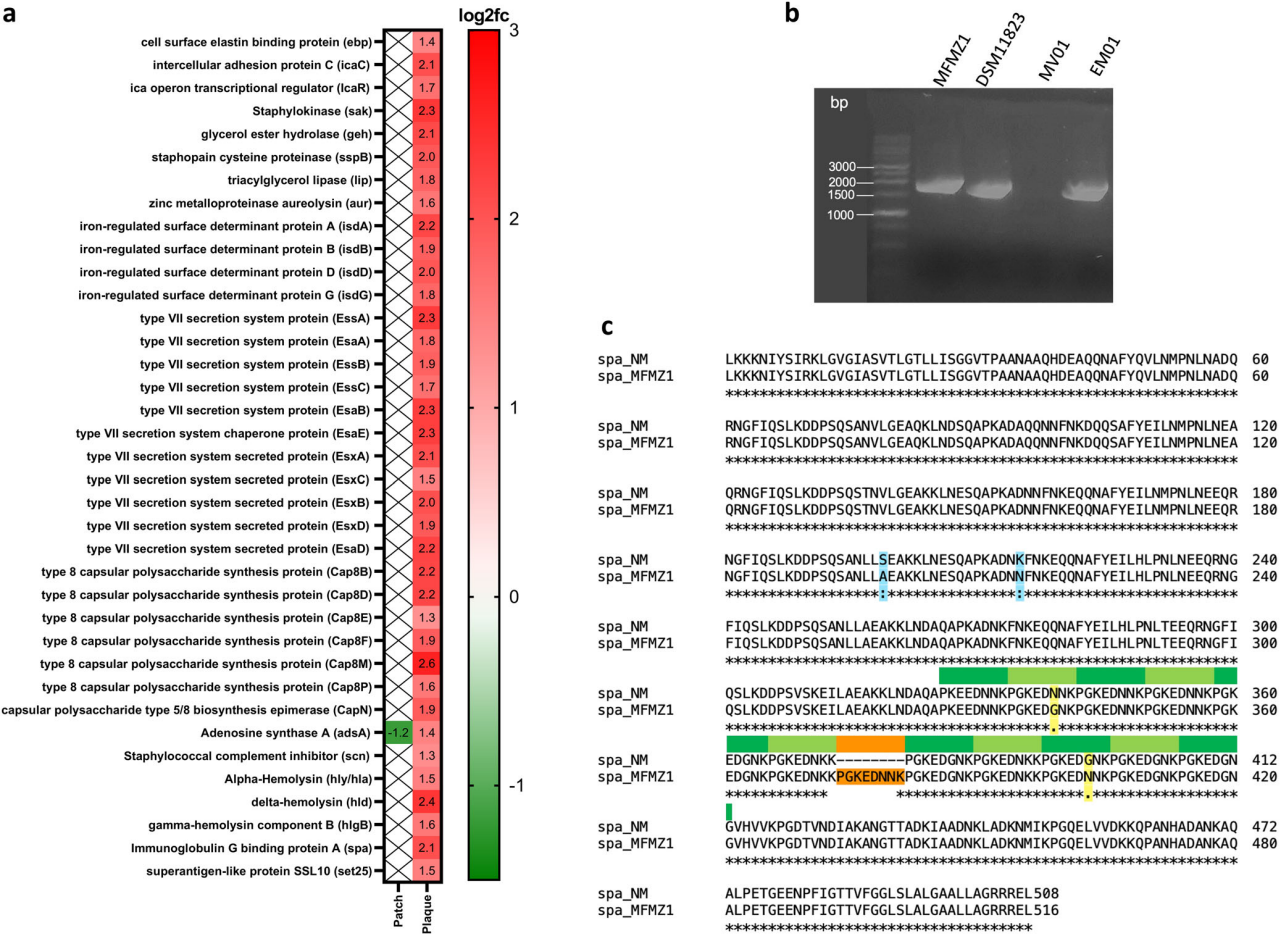
Virulence factors of the MF microbiome. **a** Differential abundance analysis of the virulence gene repertoire. Displayed are log2 fold-change (log2fc) of virolence genes on patch (n = 19) and plaque (n = 15) compaired to nonlesional (n = 31). **b** Agarose gel electrophoresis of spa genes from Staphylococci isolates after PCR amplification. From left to right: spa from S. aureus MFMZ1, *S. aureus* DSM11823 (positive control), *S. epidermidis* MV01 (negative control), and *S. aureus* EM01. All detected spa genes are located between the 1500 and 2000 bp line. **c** ClustalΩ alignment of spa from *S. aureus* MFMZ1 (spa_MFMZ1) with spa from the *S. aureus* Newman strain (spa_NM, UniProt ID: A0A0H3K686). spa_MFMZ1 was found to be 24 bp/8 aa longer than spa_NM (1551 bp/516 aa vs. 1527 bp/508 aa) due to the insertion of an additional octapeptide repeat (marked in orange) in the conserved IgG binding domain (marked in green). Furthermore, amino acid variations could be detected in the B-domain (S199A, K215N, marked light blue) and in one of the octapeptide domains of spa_MFMZ1 (N349K, G403N, marked in yellow).

Here, we found numerous virulence factors that were significantly more prevalent on plaques (Fig. 6a). Notably, the VFDB and the literature^49–52^ link these virulence factors to *S. aureus*, affirming the exclusive increased abundance of this pathogen in plaque stage. Considering that *S. aureus* was only upregulated in the ΔSA-positive subgroup (Fig. 2a, b), it is highly likely that the enriched virulence factors originated from *S. aureus* of ΔSA-positive patients.

Surprisingly, we did not find any superantigens, which are typically associated with disease progression in *S. aureus* infected CTCL patients^17,53–55^. However, we found an enrichment of α-hemolysin (*hly/hla*), another virulence factor of *S. aureus*, which is linked to MF pathogenesis^56,57^. *hly/hla* can form pores in human T cells, causing cellular damage^49^. In the context of MF, it was demonstrated that *hly/hla* preferentially induces cell death in benign T cells over malignant T cells^56^ and inhibits cytotoxic T cell mediated killing of malignant T cells^57^. Furthermore, we identified an enrichment of several virulence factors that have not been associated with MF pathogenesis before (Fig. 6a). Most of the *S*. aureus-associated genes are components of larger complexes that orchestrate nutrition, immune evasion, spread of infection and secretion of virulence factors (Supplementary Material 1 provides a comprehensive overview of these virulence factors and their functional properties). In particular, we found a differential enrichment of Immunoglubin G binding protein A (*spa*), along with iron-regulated surface determinant protein A (*isdA*) and type VII secretion system protein D (*esaD*).

Since *isdA* was reported to confer resistance to the AMP hBD2^52^, this virulence factor might have enabled *S. aureus* outgrowth on plaque despite the increased RNA-levels of *hBD2* in these lesions (Fig. 3a). An additional factor for *S. aureus* outgrowth may be *esaD*, which encodes a strong bactericidal for other bacteria than *S. aureus*, suppressing growth of competing commensals^58^.

Of particular significance was the upregulation of *spa* on plaques: As previously reported, this virulence factor can activate the NF-κB (Nuclear factor kappa-light-chain-enhancer of activated B cells) pathway via tumour-necrosis factor-α (TNF-α) receptor 1 (TNFR1) through conserved IgG binding domains^59–65^. Several studies demonstrated that the TNF-α/NF-κB pathway is dysregulated in a subset of MF patients with poor clinical outcome^29–31^, and *spa* could hence be the activating factor. We therefore next investigated *spa* in Pat1-derived isolate *S. aureus* MFMZ1 (*spa*_MFMZ1) and not only confirmed its presence within the genome (Fig. 6b), but also identified mutations via sequencing: Compared to s*pa* of the *S. aureus* Newman strain serving as a reference (*spa*_NM, UniProt ID: A0A0H3K686), *spa_*MFMZ1 had an additional octapeptide repeat (Ins373PGKEDNNK) in the conserved IgG binding domain, resulting in a total of 12 repeats (Fig. 6c). It is known that the *spa* IgG binding domain is composed of a variable number of octapeptide repeats and activates inflammatory responses via interaction with the Fc fragment of IgG^66,67^. Strains of *S. aureus* with >7 repeats are generally considered more virulent as they can bind more precisely to the Fc fragment^68,69^. Notably, the IgG binding domain of *spa* also activates TNFR1 signalling through the same octapeptide repeats^59–64^ where we detected the mutational insertion. Hence, the mutation-dependently increased number of octapeptide repeats of *spa* in the strain derived from MFMZ1 may enhance spa binding efficacy to TNFR1 and in turn pronouncedly activate the TNF-α/NF-κB axis.

In summary, these results show that *S. aureus* strains on plaque of ΔSA-positive patients were highly virulent and shaped environmental conditions to evade the host immune response, spread infection and may fuel disease progression, as deduced from the potential gain-of-function mutation of spa in the MFMZ1 strain.

### *S. aureus* assaults the T cell receptor repertoire and might promote malignancy and dissemination via spa

**Fig. 7:**
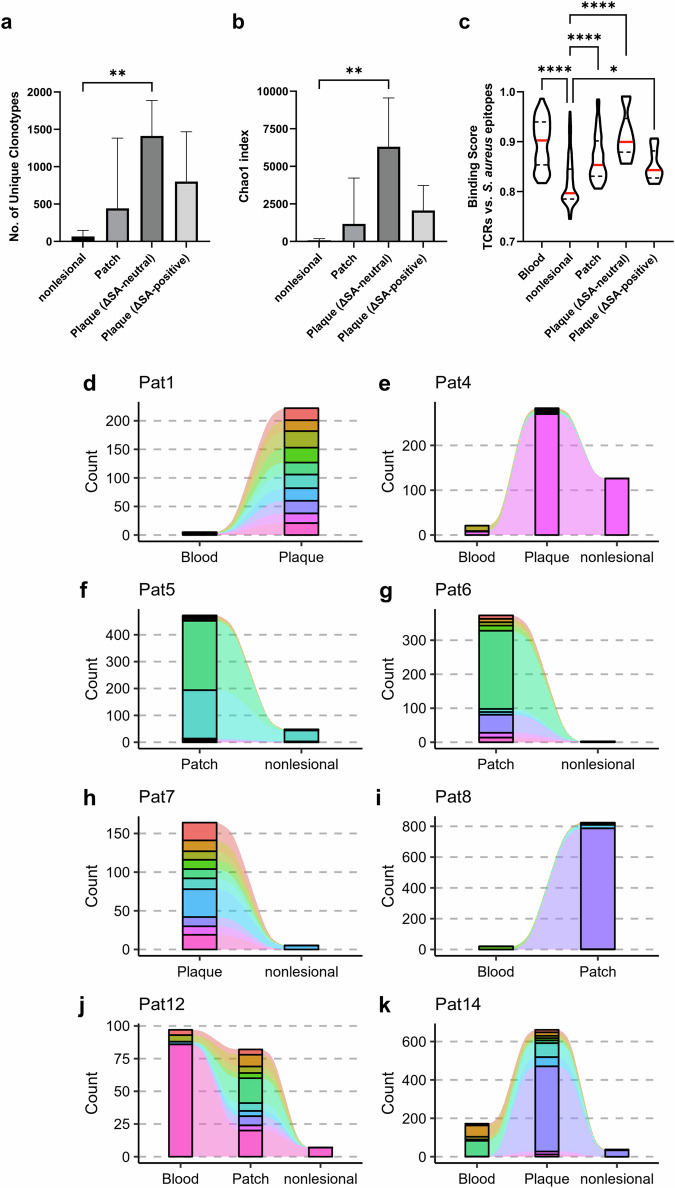
The TCR repertoire is affected by S. aureus. **a**, **b**, α-diversity of the TCR repertoire in MF lesions and nonlesional skin. Plaque samples are stratified according to ΔSA-subgroup. **c** For each given pair of TCR and *S. aureus* epitope, a Binding Score (1 = perfect binding, 0 = no binding) was calculated. Epitopes were downloaded from the IEDB or, if not available, predicted for identified virulence factors of plaque-associated S. aureus strains. n = 275, displayed are the median (red), 1^st^ and 3^rd^ quantile, Kruskal-Wallis test corrected for multiple comparisons. **d–k**, Presentation of the top abundant TCRs in MF lesions and their contribution to the TCR repertoire in nonlesional skin and blood in the same patients. Counts represent the summed abundance of TCR clones and were normalized across all patients (by subsampling to common sequencing depth). Dominant clones in MF lesions were detectable in other tissues as well.

To assess a potential impact of *S. aureus* and its identified virulence factors on the malignant and benign T cell infiltrate, we further performed T cell receptor (TCR) sequencing in the skin and blood of MF patients. Surprisingly, although one might expect a skewed TCR repertoire in MF lesions due to the expansion of malignant T cells, instead, abundance and diversity increased with lesional stage (Fig. 7a, b). This augmentation might have been caused by tumour infiltrating lymphocytes (TILs)^70^ and the expansion of more than just a single malignant clone resulting in oligoclonality^71–73^. Indeed, oligoclonality was detected in most MF lesions (Fig. 7d–k). Intriguingly, the TCR repertoire in plaques of the ΔSA-positive group was reduced (Fig. 7a, b). In addition, gene usage analysis of the T cell receptor β variable (TRBV) revealed that TRBV5-1, which another research paper linked to TILs in MF^74^, was strongly expanded in plaque of the ΔSA-negative group but not in plaque of the ΔSA-positive group (Supplementary Fig. 3). We assumed that the reduction of both total TCR repertoire as well as TRBV5-1 in plaque of ΔSA-positive patients could be due to the virulence factor hla. Hla is known to kill benign T cells in MF^56,57^, and we found this virulence factor enriched in the ΔSA-positive subgroup (Fig. 6a). Collectively, our data indicate that *S. aureus* affected the balance between malignant T cells and benign tumour infiltrating T cells.

We next examined whether T cells of MF patients were directed against the *S. aureus* virulence factors identified as enriched on plaque (Fig. 6a). Corresponding epitopes were obtained from the Immune Epitope Database (IEDB)^75^, or, when not available in the IEDB, computationally predicted for both Major Histocompatibility Complex class I (MHC-I) and II (MHC-II). Binding Scores were calculated for the most abundant TCRs of a given sample and each of the epitopes (obtained and predicted) of *S. aureus* virulence factors. As expected, TCRs in patch, plaque and blood showed a significantly higher affinity for *S. aureus* virulence factors compared to TCRs in nonlesional skin (Fig. 7c). The increased binding affinity in blood and patch might be due to recirculation of benign skin resident T cells being called to sites of inflammation^76,77^ as well as the recently described effect of clonal seeding by malignant T cells^71,73,78^. Analogues to the TCR repertoire, the binding affinity of TCRs in ΔSA-positive plaques was lower compared to ΔSA-neutral plaques, probably caused by the virulence factor hla which preferentially kills benign reactive infiltrate over malignant T cells^56,57^. Notably, dominant T cell clones of MF lesions were also detected in nonlesional skin and the blood (Fig. 7d–k). In all tissues tested, the MHC-II epitope of spa was among the epitopes that was recognized most often by TCRs (Supplementary Fig. 4). MHC-II interacts with the TCR of CD4 + T cells, which is the T cell subset that undergoes malignant transformation in MF^79^. As already outlined above, spa is known to activate NF-κB and to trigger inflammation^59,60,63–65^, typical characteristics of progressive MF^2^. Collectively, our results indicate that the plaque-enriched *S. aureus* virulence factors, and of these especially spa, were recognized by T cells in MF lesions and that those clones might disseminate into other tissue compartments, thus driving pathogenesis.

### ΔSA-positive patients have an inferior event-free survival

We demonstrated that *S. aureus* strains on MF lesions differed from nonlesional counterparts being highly virulent, and dominated the plaque microbiome of ΔSA-positive patients, which had a comprised TCR repertoire that specifically recognized *S. aureus* virulence factors. Hence, the ΔSA-positive subgroup might undergo a more severe course of disease. Therefore, the clinical response of the MF study cohort was monitored while patients were treated with approved MF therapy adequate to the patients’ individual situation. Event-free survival (EFS) was evaluated using Kaplan-Meier analysis. Events were defined as death, start of new therapy and disease progression. Two out of 20 patients were lost to follow-up (LFU) for clinical assessment and therefore excluded from analysis. 9 out of 18 patients experienced an event during the observation period (2.5–18.4 months, median 11.5). Metrics are summarized in Table 1.

**Fig. 8:**
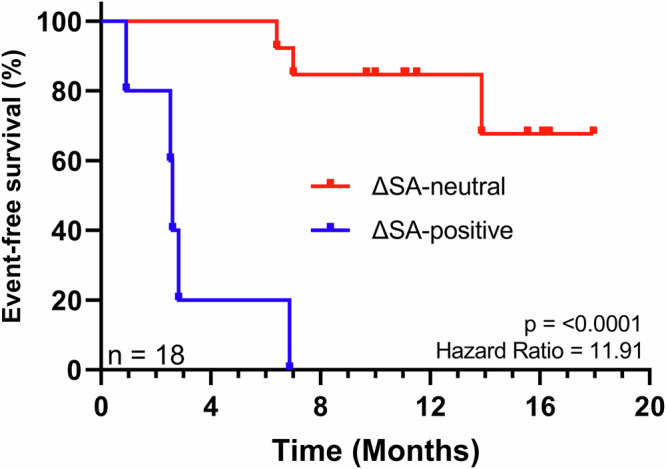
Event-free survival Kaplan-Meier curves. Patient Enrolment started in September 2020 and was completed in April 2021. The follow-up period lasted until May 2022. The Log-rank test was used to determine differences between survival curves (1 degree of freedom).

Patients in the ΔSA-positive group exhibited a strikingly inferior EFS with a hazard ratio of 11.91 (95% confidence interval 1.44 to 98.36) (Fig. 8). Median EFS was 2.6 months in the ΔSA-positive group and not reached in the ΔSA-neutral group. No EFS associations were found for age and gender, however, EFS was significantly lower for patients treated systemically and when undergoing radiotherapy, respectively (Supplementary Fig. 5a, b). This is to be expected as these lines of therapy are chosen in advanced-stage or refractory early-stage MF^80^, and are thus associated with poor clinical course^4^. The cohort analysed consists of 14 patients in early-stage (IA-IIA) MF and 4 patients in advanced-stage (IIB) MF. Since advanced-stage MF tends to progress faster than early-stages^4^, joining clinical stages should be considered with caution. Notably, the four 4 stage IIB MF patients were distributed equally between the ΔSA-subgroups (two each) and independent Kaplan-Meier analysis of early- and advanced stage MF patients yielded similar results (Supplementary Fig. 5d, e) as the joint analysis depicted in Fig. 8.

This demonstrates that the skin-microbiome stratifies MF patients into two subgroups with distinct clinical outcomes, opening-up the potential of new treatment regimens.

## Discussion

An increasing body of evidence demonstrated the profound impact of human body colonizing microbes on the cancer disease course, especially enabled by the advent of new sequencing technologies^81^. While the gut microbiome has been in the focus of investigations, the skin microbiome is increasingly recognized for its role in modulating the cutaneous immune system and its resulting implications for several inflammatory and neoplastic skin conditions^82^. A convincing body of evidence proposes the influence of single bacterial agents, especially *S. aureus*, on MF pathogenesis^14–20^. However, *S. aureus* is also a physiological commensal on healthy skin^21^ and not every MF patient was found to be skin colonized by this species^17,18^. Additionally, while systemic or topical antibiotic treatment successfully eliminated *S. aureus* in the majority of culture-positive MF patients, only 53% – 58% responded to therapy^18,22^.

In this study, we investigated the role of the skin microbiome in 20 MF patients using metagenomic sequencing. We obtained several findings that give novel insights into the pathophysiological role of the skin microbiome for MF progression along with potential mechanisms that underly these distinct changes:

First, we were able to identify microbiome patterns that stratified our patient cohort into the two subgroups ΔSA-positive and ΔSA-neutral. In the ΔSA-positive subgroup, the relative abundance of *S. aureus* increased from nonlesional skin to plaque, a significant dysbiosis existed on plaque, the TCR repertoire was assaulted and EFS was strongly decreased.

Second, we observed an overexpression of AMPs in MF lesions as compared to nonlesional skin, which is in accordance with previous reports^83–85^. In addition, our results revealed that clinical isolates of MF skin commensals were more resistant to several of these AMPs as compared to microbial isolates from healthy skin. Notably, the AMP hBD1 even augmented growth of plaque-derived *S. aureus* (Fig. 3c), probably acting like a co-factor. Therefore, we reasoned that the sustained and sublytic expression of AMPs might initially eradicate the physiological skin flora while, eventually, some commensals adopt to the new conditions and develop resistances, allowing them to live under ectopic AMP levels of MF skin. Besides this indirect disease promoting effect, AMPs may also directly drive MF progression. In particular, AMPs were reported to recruit CD4^+^ (memory) T cells^86,87^, which represent the neoplastic T cell subtype in MF^79^. This interaction might result in a positive feedback loop in which the production of AMPs recruits neoplastic T cells, that enhance the inflammatory microenvironment and augment AMP levels. Although AMP expression levels were increased in MF lesions, they did not differ between ΔSA-positive and ΔSA-neutral patients. Thus, enhanced AMP levels might not explain the molecular and clinical differences of the two patient subgroups.

Rather, this study identified that *S. aureus* strains on plaques of the ΔSA-positive subgroup were distinct from their nonlesional counterparts, displaying a unique gene repertoire and being considerably more resistant towards commonly used antibiotics than all other tested bacteria. Further, we identified several virulence genes that were enriched in the plaque microbiome of ΔSA-positive patients. Interestingly, our analysis did not reveal the presence of staphylococcal superantigens within the pool of virulence genes, which have been regarded as a significant factor in *S. aureus*-dependent MF progression so far^17,53–55^. Instead, our attention was drawn to the virulence factor *spa*. It has been shown that spa activates the NF-κB pathway and induces inflammation by serving as ligand for the TNFR1^59–65^. Also, it was reported that *S. aureus* triggers the release of TNF-α in keratinocytes^88^, which in turn may activate NF-κB in malignant T cells in a paracrine fashion. In line, several studies showed that the NF-κB pathway is recurrently deregulated in a subset of MF patients with an inferior disease course^29–31,89^. Notably, our results revealed that *spa* of the plaque-derived *S. aureus* isolate MFMZ1 contained a potential gain-of-function mutation that may render it even more potent in activating the TNFR1/NF-κB axis^59,60,68,69,90^. Therefore, stimulation of the NF-κB pathway by spa could account for the inferior EFS of the ΔSA-positive subgroup. Consistent with these findings, our results from TCRseq demonstrated that plaque-enriched *S. aureus* virulence factors, and spa in particular, were recognized by T cells in MF lesions. Given that those clones also presented in blood and nonlesional skin, indicates that these specific T cell clones might disseminate into other tissue compartments, thus driving pathogenesis.

Collectively, our results provide a strong rationale for treating MF patients with microbiome-modulating drugs. Since we showed that the presence of *S. aureus* alone is not necessarily associated with an aggravated disease course, we suggest stratifying MF patients into ΔSA-subgroups rather than subjecting all *S*. aureus-positive MF patients to antibiotic therapy^18,22^. In addition, more precise microbiome-modulating drugs are warranted because antibiotics also eradicate the physiological skin flora and provoke microbial resistances^28,91^. An appropriate therapeutic approach could be bacteriotherapy by transferring the microbiome or a single bacterial species of nonlesional skin onto MF lesions. In previous reports, *S. epidermis* was found to inhibit growth of malignant cells in skin neoplasms in vitro and in vivo while sparing normal keratinocytes^92^. Moreover, a clinical phase 1 trial showed that patients with atopic dermatitis (AD) benefit from re-introduction of an *S. hominis* strain onto AD lesions that was isolated from nonlesional AD skin^93^. AD and MF share some molecular characteristics of the inflammatory microenvironment^94,95^ and, moreover, some MF microbiome patterns identified by us in the present study were also observed in AD. In particular, AD severity is associated with an increase of *S. aureus*, concomitant α-diversity decrease^96^, and distinct *S. aureus* strains between AD lesions and nonlesional skin^97^. Thus, re-introduction of *S. hominis* on MF plaque may be a therapeutic option. In addition, our results suggest that *S. epidermidis*, *S. hominis* and *C. acnes* could help to maintain stable disease in MF patients as those commensals were significantly counterbalanced to *S. aureus* in the ΔSA-neutral group exhibiting fewer progressive events. In line, all three commensals were found beneficial for protection against *S. aureus* in clinical settings^36–39^.

Limitations of our study are the small patient cohort, the location of the two study centres that lie within the same geographic area which might add a selection bias to the *S. aureus* strains^98^, the small amount of patients from which clinical microbial species were isolated, and the relatively short follow-up period for the usually indolent course of MF. Furthermore, the lack of a healthy control group would have allowed for more definitive conclusions on the composition and the functional properties of microbes found in nonlesional skin of MF patients. In particular, our analysis could not identify a specific factor or event that facilitated *S. aureus* outgrowth on plaques of ΔSA-positive patients, after most of the normal skin flora is eradicated upon the onset of aberrant AMP expression. Since we demonstrated that lesional *S. aureus* strains differed from their nonlesional counterparts, it may be possible that ΔSA-positive patients were overgrown with this virulent *S. aureus* strain during a period of decreased microbial defence barrier, opening a “window of opportunity”. In turn, ΔSA-neutral patients did not experience such an event and their skin microbiome was able to recover, (re-)building an intact microbial defence barrier. Additional research involving larger patient cohorts, an expanded range of clinical bacterial isolates, and functional assays is required to elucidate the underlying cause of *S. aureus* overgrowth in the ΔSA- positive subgroup.

Also, we cannot definitively determine whether the disturbance in the microbiome was a consequence of our proposed mechanism, where initial infiltration of malignant T cells led to ectopic AMP expression, or if the skin was rather initially infected with a virulent and AMP-resistant *S. aureus* strain, triggering ectopic AMP levels resulting in secondarily acquired dysbiosis. Of note, *S. aureus* was more abundant on nonlesional skin of ΔSA-positive patients (Fig. 2b), which would support the alternative hypothesis at first sight. In contrast, our results indicate that the lesional *S. aureus* strain may have spread into nonlesional areas of the skin, as suggested by two ΔSA-positive patients with the same virulent *S. aureus* strain present on both plaque and nonlesional skin (Fig. 4a). In line, it was shown that MF patients have a comprised physical skin barrier even in nonlesional skin, rendering them more susceptible to infections^83,84^.

Open issues also remain about the role of *C. acnes* in MF pathogenesis. While this species displays anti-*S. aureus*-activity^36^, C*. acnes* also is an opportunistic pathogen in various infectious diseases and induces secondary aggregation as well as biofilm formation of *S. aureus*^99,100^. In addition, *C. acnes* can cause inflammation, which is a chronic condition in MF and boosts progression^1,2,99^. Linking *C. acnes* to the pathogenesis of (early-stage) MF seems therefore plausible. Indeed, *C. acnes* was highly abundant on patches of ΔSA-positive patients (Fig. 2f). Yet, our results involved only two data points and *C. acnes* was upregulated in some patches of ΔSA-neutral patients as well (Fig. 2f). Further research is therefore warranted to understand if *C. acnes* plays a role in MF pathogenesis.

In summary, our study offers comprehensive insights into the skin microbiome of MF patients and identified dysregulation of the skin microbiome and concomitant outgrowth of virulent *S. aureus* strains as critical components that can stratify MF patients into distinct subgroups of MF severity. The microbiome perturbations might be caused by an aberrant AMP production, enabling the outgrowth of AMP-resistant *S. aureus*. Strains of this pathogen harboured several virulent properties, most notably the virulence factor *spa*, which likely drove the pathogenesis in the ΔSA-positive subgroup with inferior prognosis. Although our study is exploratory in its nature, our data provide a strong rational to treat the skin of MF patients with drugs that either reconstitute a physiological skin flora or build up epithelial integrity. One possibility could be bacteriotherapy with commensals that we identified being counterbalanced to *S. aureus*. Further studies are needed to explore the underlying mechanism why one subgroup experiences *S. aureus* outgrowth while the other subgroup is spared, if *S. aureus* activates NF-κB via spa to fuel MF progression, and to delineate the role of other commensals such as *C. acnes* in MF progression.

## Methods

### Patient recruitment and clinical specimens

Patients were recruited from two skin cancer centres in Germany, the Department of Dermatology, University Medical Centre Mainz, and the Department of Dermatology, Venerology and Allergology, University Hospital Frankfurt am Main. Eligibility criteria included diagnosis of MF, aged between 18 and 90 years, no antibiotic treatment at a minimum of 1 month prior to sampling, no showering or application of cremes and antiseptic agents at a minimum of 24 h prior to sampling. Patients with allergies against local anaesthetics were excluded from skin biopsies. The following clinical specimens were taken, all at the same visit: Microbiome swabs from lesional and nonlesional skin, skin punch biopsies from the same lesions and nonlesional skin, peripheral blood.

### Microbiome sampling and DNA extraction

We sampled up to two separate lesions from each patient. To account for the high individuality of microbiome profiles^21,101^, we included intra-patient controls instead of other subjects with e.g., healthy skin or patients with skin conditions other than MF. To this end, we collected nonlesional skin samples from the contralateral site of lesional samples of the same MF patient.

procedure and DNA extraction was performed as described previously with a few modifications^21,24,102^. A swab-scrape-swab procedure was used to sample microbes from the skin. Yeast cell lysis buffer from the MasterPure Yeast DNA Purification Kit (MPY80200, Lucigen, USA) was aliquoted into individual collection tubes holding baskets with a semi-permeable valve (NAO™ Basket 4103CS01, COPAN, USA). A sterile swab (FLOQSwab 4N6, NAO™ Basket 4103CS01, COPAN, USA) pre-moistened in the aliquoted buffer was brushed vigorously over the skin and placed back into the collection tube holding the basket and buffer. Then, a surgical scalpel was carefully scraped over the same skin area and placed into the tube holding the buffer-containing basket. This was followed by brushing the skin with the same swab used in the first step. The swab was placed into the collection tube and the swab head was clipped off. The collection tube was directly placed on dry ice and stored at −80 °C until DNA extraction. The whole sampling procedure was performed wearing sterile gloves and surgical masks.

To extract the DNA, frozen samples were first thawed at 37 °C for 10 min on a heated shaking block. Samples were then treated with 1 μl ReadyLyse Lysozyme solution (MGP04100, Lucigen, USA) at 37 °C for 60 min on a heated shaking block. After incubation, the sample solution was separated from the swab head with the help of the semi-permeable valve in the baskets by centrifuging at 12,000 g for 2 min at room temperature. The basket containing the swab head was discarded. Then, beads (Pathogen Lysis Tubes, 19092, Qiagen, Germany) were added into the collection tube to mechanically disrupt cells using a TissueLyser II (85300, Qiagen, Germany) for 10 min at 30 Hz. Samples were then incubated at 67 °C for 30 min and placed on ice for 5 min. After adding 150 μl MPC precipitation reagent from the MasterPure Yeast DNA Purification Kit, cell debris was spun down and the supernatant was transferred to a fresh DNA LoBind Tube (0030108051, Eppendorf, Germany). Samples were combined with an equal volume of 100% ethanol (15420665, Fisher BioReagents, USA) and purified using the PureLink Genomic DNA Mini Kit (K182002, Invitrogen, USA) according to the manufacturer’s instruction. Finally, DNA was eluted in 25 μl 0.1 TE.

### Preparation of metagenomic libraries and Whole Metagenome Sequencing

Illumina sequencing libraries were created using the NEBNext Ultra II FS DNA Library Prep Kit (E7805L, New England Biolabs, USA) in combination with NEBNext Multiplex Oligos, Dual Index Set 1 (E7600S, New England Biolabs, USA) according to the manufacturer’s instructions. Library qualities were assessed with the 2100 Bioanalyzer (G2939BA, Agilent, USA) and Qubit 2.0 (Q32866, Invitrogen, USA). Whole Metagenome sequencing was performed on Illumina NovaSeq 6000 at 150 bp paired-end to an average of 47,519 million raw reads (7.13 Gbp) per sample.

To control for consistency in DNA extraction and metagenomic library preparation, we included a standardized mock community of inactivated microbial cells (ZymoBIOMICS Microbial Community Standard, Zymo Research, USA) and thereof derived microbial DNA (ZymoBIOMICS Microbial Community DNA Standard, Zymo Research, USA) that we processed and sequenced alongside the MF-metagenomic samples. Clustering analysis of the microbial community profiles obtained from both standards revealed high similarity between each processing batch and concordance with the manufacturer’s specifications (Supplementary Fig. 6).

### Metagenomic profiling

Preparation steps and analysis of metagenomic samples were conducted using the bioBakery toolsuit^103^. Raw reads were first quality checked, trimmed and human reads were filtered out with KneadData version 0.7.10, which integrates the tools FastQC 0.11.9^104^, Trimmomatic 0.33^105^, and Bowtie2 2.4.1^106^. An average of 1,312 million non-human, quality-filtered reads (0.34 Gbp) were obtained per sample and used for all subsequent steps. More non-human reads were retained from nonlesional skin compared to MF lesions, probably due to the nature of MF lesions being scaly^2^. In consequence a greater proportion of human skin cells were sampled from MF lesions compared to nonlesional skin. A similar effect was observed in a study investigating the skin microbiome of Psoriasis using a similar analysis pipeline^107^.

Taxonomic profiles were generated with MetaPhlAn 3.0.2^103,108^ successfully for a total of *N* = 65 metagenomic samples. Relative abundances of each clade were obtained with the flag –analysis_type rel_ab. Taxonomic tables of each sample were merged with the merge_metaphlan_tables.py script and only the species level retained. To profile virulence genes, protein sequences were obtained from the Virulence Factor Database (VFDB)^48^. These were used as input to the Identify-mode of ShortBRED^47^. Briefly, ShortBRED clusters proteins of interest into families and finds markers for every built family that are unique against an exhaustive database of proteins (UniRef90). Then, ShortBRED-Quantify uses translated search to quantify the identified protein markers in metagenomic reads and normalizes results to produce a functional profile. Differential abundance analyses were conducted with MaAsLin 2^35^ building a generalized linear mixed model, with stage being the dependent variable and patient and number of non-human reads as co-variates. Relative abundances were log-scaled and nonlesional skin was set to reference level for the dependent variable (i.e., stage). Other settings were left to default.

Strain profiling was performed with PanPhlAn 3.1^46^. The tool characterizes the gene composition of individual strains by mapping metagenomic samples against the pangenome of a species of interest. For profiling, the least stringent option was carried out by adding respective flags as described in the manual (--min_coverage 1 --left_max 1.70 --right_min 0.30). The resulting binary table showing presence/absence of genes was loaded into R! and a PCA was performed with the internal prcomp function. Plots were generated with GraphPad PRISM 9 and ggplot2^109^.

### Diversity analysis and clustering of taxonomic profiles from rarefied metagenomic samples

Diversity metrics and clustering analysis was carried out from metagenomic reads rarefied to common depth. To generate rarefaction curves, non-human reads were subsampled to increasing depths using seqkit 0.15.0^110^ with subsequent taxonomic profiling. Rarefaction curves resembled those of another study investigating the skin microbiome of psoriasis patients using a similar analysis pipeline^107^. Generally, more unique species were observed on nonlesional skin than on MF lesions (Supplementary Fig. 7). This could be associated with the higher sequencing depth of nonlesional skin. Yet, also at a given subsampled depth nonlesional skin presented with more unique microbial species, showing that the differences are rather of a biological than a technical nature^107^. For diversity analysis and clustering of taxonomic profiles, metagenomic reads were subsampled to 141,703 reads. Lesional skin samples tended to have less non-human reads and thus more lesional samples were filtered out by rarefaction compared to nonlesional samples. Thus, to maintain the intrapatient sample-control regimen, nonlesional samples without a counterpart from lesional skin were filtered out, resulting in a total of 32 samples for subsequent analyses. Shannon-Index and Bray-Curtis dissimilarities were calculated with the R! package vegan^111^. The same package was also used to calculate PERMANOVA with the adonis function as well as PERMDISP with the betadisper function. A statistically significant signal from adonis was only considered trustworthy with concomitant non-significant signal from betadisper to rule out inhomogeneity of dispersion among groups. Pairwise comparisons were performed with the R! package pairwiseAdonis^112^. Clustering and plotting of taxonomic profiles were done with the R! package pheatmap.

### Skin biopsies, peripheral blood, and RNA extraction

4 mm punch biopsies were obtained from the same lesions sampled for metagenomics and at the same visit. The punch biopsies were immediately shock frozen and kept in liquid nitrogen until processed. RNA was extracted using the RNeasy Fibrous Tissue Mini Kit (74704, Qiagen, Germany) along with the TissueLyser II (85300, Qiagen, Germany) according to the manufacturer’s instructions. Peripheral blood mononuclear cells (PBMCs) were isolated from freshly drawn blood using density gradient centrifugation (Histopaque-1077, SIGMA-ALDRICH, Germany) and kept in liquid nitrogen until processed. PBMCs were thawed at 37 °C, washed in pre-warmed RPMI 1640 medium (31870-025, Gibco, USA) including 10% FCS and counted using the Countess 3 machine (ThermoFisher Scientific, USA). To enrich the T cell population, the Pan T Cell Isolation Kit human (Miltenyi Biotec, Germany) was used according to the manufacturer’s instructions. Directly thereafter total RNA was extracted with the RNeasy Plus Mini Kit (Qiagen, Germany) according to the manufacturer’s instructions.

### RT-qPCR of AMPs

First, synthesize of first strand cDNA was carried out with M-MuLV Reverse Transcriptase (M0253L) using both Random Hexamer Primer (S1330S) and Oligo(dT) Primer (S1316S) (all from New England Biolabs, USA) at a total reaction volume of 20 µl and incubated as noted in the manual. Final cDNA was quantified on a NanoDrop 2000c (ND-2000, ThermoFisher Scientific, USA). For RT-qPCR the primaQUANT SYBRGreen Master Mix (SL-9902HRB, Steinbrenner Laborsysteme, Germany) was used with 400 ng template as input at a total reaction volume of 25 µl. The 7300 Real-Time PCR System (Applied Biosystems, USA) was used with an initial denaturation at 95 °C for 10 min followed by 40 cycles of 95 °C for 15 s and 60 °C for 1 min. The runs were evaluated via melting curves. Primers for AMPs and housekeeping gene were used from literature or self-designed and are listed in Supplementary Table 2.

### T cell receptor sequencing and analysis

Illumina sequencing libraries were constructed with the NEBNext Immune Sequencing Kit, human (E6320, New England Biolabs, USA) according to the manufacturer’s instructions with 200 ng total RNA as starting material. The optimal number of PCR cycles for target amplification was determined for each sample by RT-qPCR as outlined in the manual. Final libraries were quality checked via Qubit 2.0 (Q32866, Invitrogen, USA) and the 2100 Bioanalyzer (G2939BA, Agilent, USA). Because of the low T cell proportion especially in nonlesional skin, samples with inappropriate TCR libraries were excluded from further analysis. A total of 21 libraries (16 from MF skin, 5 from PBMCs enriched for T cells) were pooled and then sequenced on a MiSeq (Illumina, USA) with 10% PhiX. Sequencing was performed running 300 bp PE using a 600-cycle V3 reagent kit to an average of 0.98 million reads per sample.

The sequencing data were uploaded to the Galaxy web platform and processed with the pRESTO toolkit^113^ implemented in a publicity available workflow at https://usegalaxy.org/u/bradlanghorst/w/presto-nebnext-immune-seq-workflow-v320 using default parameters. Subsequently, the R toolkit immunarch 0.7.0 was used to analyse the TCR repertoire and to visualize T cell clonotype tracking as well as TCR gene usage^114^. To investigate if TCRs recognize *S. aureus* virulence factors that we identified as enriched on plaque, we downloaded corresponding epitopes from the IEDB (https://www.iedb.org/)^75^. If an epitope of was not available, it was predicted with an online tool for both MHC-II and MHC-I (http://tools.iedb.org/main/tcell/). A table was constructed containing pairs of the most abundant TCR clones with each epitope (i.e., epitopes received from the IEDB and epitopes predicted with the online tool). The python tool ERGO-II was used to calculate a binding score of each TCR-epitope pair. The higher the binding score, the higher the probability that the TCR recognizes and binds the epitope^115^.

Although plaque samples of Pat1 and Pat7 did not show monoclonal expansion according to our RNA-based TCR sequencing approach, we confirmed the diagnosis of MF during the further clinical course: Pat1 developed a tumour (T2b, >7 cm diameter), and several patch and plaque lesions. Regarding Pat7, we detected T cell clonality against a polyclonal background using the clinical standard-of-care BIOMED-2 protocol. BIOMED-2 is a DNA-based TCR sequencing approach^116^, whereas we performed RNA-based TCR sequencing. Both methods differ in several aspects, e.g., RNA-based TCR sequencing has a higher sensitivity and detects more TCR clones^117,118^.

### Isolation of clinical microbial species and functional analyses

Clinical isolates of microbial species were obtained from the same MF patients enroled in the metagenomic survey. The skin was swabbed several times, spread on a *Staphylococcus* Vogel-Johnson agar [1% (w/v) casein peptone, 1% (w/v) mannitol, 1% (w/v) glycine, 0.5% (w/v) yeast extract, 0.5% (w/v) lithium chloride, 0.5% (w/v) dipotassium phosphate, 0.0025% (w/v) phenol red, 0.02% (v/v) potassium tellurite, 2% (w/v) agar, pH 7.2]^119,120^, and samples were incubated at 37 °C overnight. *S. aureus* colonies should appear small, black surrounded by a green-yellow zone, while *S. epidermidis* strains appear grey-black without zones. Putative *S. aureus* and *S. epidermidis* colonies were picked and checked via colony PCR using *Staphylococcus tuf* specific primers (TStaG422: GGCCGTGTTGAACGTGGTCAAATCA & TStag765: TIACCATTTCAGTACCTTCTGGTAA) according to^121^. The achieved fragments were sequenced to identify the *Staphylococcus* strain.

*Antibiotic diffusion assay* was performed to determine antibiotic resistances of clinical *Staphylococci* isolates. Therefore, 100 µl of an overnight culture were pipetted into 20 ml melted 0.8% semi solid HD agar (w/v) and poured into petri dishes. After solidification, 5 antibiotics (25 µg ampicillin, 100 µg carbenicillin, 23.75 µg sulfomethoxazol + 1.25 µg trimetophrim, 15 µg erythromycin, and 5 µg novobiocin) were applied using Sensi-Disc™ BD dispenser. Furthermore, resistance/sensitivity towards an additional antibiotic, methicillin, was separately tested applying 5 µg and 20 µg on sterile filter disks. The plates were kept for 2 h at 4 °C to allow antibiotics to diffuse. Afterwards the plates were incubated at 37 °C for 24 h. Antibiotic sensitivity became visible as degradation zone around the filter disks. Halo size was measured using ImageJ.

*Antibacterial assay* was performed to test whether the clinical isolates exhibit resistance towards selected AMPs, as described in^122^. Overnight cultures of bacterial strains were harvested, washed in 1x PBS [137 mM NaCl, 2.7 mM KCl, 10 mM Na_2_HPO_4_, 1.8 mM KH_2_PO_4_, pH 7.2], and resuspended in 10 mM sodium phosphate buffer (NaPi; pH 6.8). Afterwards, 10 µl of a 10^6^ cells/ml bacterial suspension were inoculated into 190 µl of 10 mM sodium phosphate buffer (10^4^ cells in 200 µl final volume) w/o various AMP concentrations (1 and 5 µg) and incubated for 2 h at 37 °C. 100 µl of the reaction mixture were plated on TSB agar plates [peptone/tryptone 1.7% (w/v), soy peptone 0.3% (w/v), D(+)-glucose 0.25% (w/v), NaCl 0.5% (w/v), K2HPO4 0.25% (w/v), meat extract 0.3% (w/v), pH 7.3] and then incubated at 37 °C overnight. The number of CFU was determined and the antibacterial effect was assessed by determining survival rate of AMP-treated *S. aureus* compared to the total number of cells in the control experiment. Three biological replicates were tested.

*Identification and sequencing of spa* was performed to determine whether the *spa* gene was present in clinical isolates. Therefore, *spa-*specific primers (Supplementary Table 2) were designed for gene amplification and PCR was performed using Q5® high-fidelity DNA polymerase from NEB according to the manufacturers protocol. As a control, PCR was performed using *S. aureus* EM01 and *S. epidermidis* MV01 obtained from the two healthy patients enroled in this study. After detecting positive amplicons via 1.5% (w/v) agarose gel electrophoresis and purification with „*HiYield*® PCR Clean-Up & Gel-Extraction Kit” (SLG) the genes were sequenced through the Sanger method^123^ by StarSeq® GmbH (Mainz). For sequencing the respective primers for *spa* were used. For more accurate result the sample was further sequenced using an additional primer *spa*_mid: cttaaaagatgacccaagcc binding in the middle of the gene. Sequencing data were analyzed, and alignments of gene and amino acid sequences were performed using Benchling (https://www.benchling.com/) and the online implementation of ClustalΩ (ref.^124^, https://www.ebi.ac.uk/Tools/msa/clustalo/) using default parameters.

### Reporting summary

Further information on research design is available in the Nature Research Reporting Summary linked to this article.

### Supplementary information

Reporting Summary

Suppmenental Material 1

## Data Availability

All relevant data are available from the authors: WMS Sequencing data and associated analysis files can be accessed at the Gene Expression Omnibus (GEO) under GSE221149 (https://www.ncbi.nlm.nih.gov/geo/query/acc.cgi?acc=GSE221149). TCR Sequencing data and associated analysis files can be accessed under GSE218874 (https://www.ncbi.nlm.nih.gov/geo/query/acc.cgi?acc=GSE218874). Both are part of the SuperSeries GSE221150 (https://www.ncbi.nlm.nih.gov/geo/query/acc.cgi?acc=GSE221150).

## Code availability

The code used for the analyses can be found at https://github.com/phlicht/Code_for_analyses_and_graphs/tree/main.
