# Supplementary material for: The skin microbiome stratifies patients with cutaneous T cell lymphoma and determines event-free survival
Source: NPJ Biofilms Microbiomes. 2024 Aug 29;10:74. doi: 10.1038/s41522-024-00542-4 (PMC11358159; doi:10.1038/s41522-024-00542-4)
Supplement: Supplementary file 1 — Reporting Summary [file 41522_2024_542_MOESM1_ESM.pdf]

nature portfolio

Corresponding author(s): Volker Mailänder

Last updated by author(s): 30.04.2024

Reporting Summary

Nature Portfolio wishes to improve the reproducibility of the work that we publish. This form provides structure for consistency and transparency in reporting. For further information on Nature Portfolio policies, see our Editorial Policies and the Editorial Policy Checklist.

Statistics

For all statistical analyses, confirm that the following items are present in the figure legend, table legend, main text, or Methods section.

n/a	Confirmed	
☐	☒	The exact sample size (*n*) for each experimental group/condition, given as a discrete number and unit of measurement
☐	☒	A statement on whether measurements were taken from distinct samples or whether the same sample was measured repeatedly
☐	☒	The statistical test(s) used AND whether they are one- or two-sided<br>*Only common tests should be described solely by name; describe more complex techniques in the Methods section.*
☐	☒	A description of all covariates tested
☐	☒	A description of any assumptions or corrections, such as tests of normality and adjustment for multiple comparisons
☐	☒	A full description of the statistical parameters including central tendency (e.g. means) or other basic estimates (e.g. regression coefficient) AND variation (e.g. standard deviation) or associated estimates of uncertainty (e.g. confidence intervals)
☐	☒	For null hypothesis testing, the test statistic (e.g. *F*, *t*, *r*) with confidence intervals, effect sizes, degrees of freedom and *P* value noted<br>*Give P values as exact values whenever suitable.*
☒	☐	For Bayesian analysis, information on the choice of priors and Markov chain Monte Carlo settings
☒	☐	For hierarchical and complex designs, identification of the appropriate level for tests and full reporting of outcomes
☒	☐	Estimates of effect sizes (e.g. Cohen's *d*, Pearson's *r*), indicating how they were calculated

Our web collection on statistics for biologists contains articles on many of the points above.

Software and code

Policy information about availability of computer code

Data collection	As this study is based on a variety of software packages we refer here to the detailed Materials and Methods section.
Data analysis	The code used for the analyses can be found at https://github.com/phlicht/Code_for_analyses_and_graphs/tree/main.

For manuscripts utilizing custom algorithms or software that are central to the research but not yet described in published literature, software must be made available to editors and reviewers. We strongly encourage code deposition in a community repository (e.g. GitHub). See the Nature Portfolio guidelines for submitting code & software for further information.

Data

Policy information about availability of data

All manuscripts must include a data availability statement. This statement should provide the following information, where applicable:

- Accession codes, unique identifiers, or web links for publicly available datasets
- A description of any restrictions on data availability
- For clinical datasets or third party data, please ensure that the statement adheres to our policy

WMS Sequencing data and associated analysis files can be accessed at the Gene Expression Omnibus (GEO) under GSE221149 (https://www.ncbi.nlm.nih.gov/geo/query/acc.cgi?acc=GSE221149). TCR Sequencing data and associated analysis files can be accessed under GSE218874 (https://www.ncbi.nlm.nih.gov/geo/query/acc.cgi?acc=GSE218874). Both are part of the SuperSeries GSE221150 (https://www.ncbi.nlm.nih.gov/geo/query/acc.cgi?acc=GSE221150).

Research involving human participants, their data, or biological material

Policy information about studies with human participants or human data. See also policy information about sex, gender (identity/presentation), and sexual orientation and race, ethnicity and racism.

Reporting on sex and gender	Gender is reported throughout the manuscript.
Reporting on race, ethnicity, or other socially relevant groupings	not reported here.
Population characteristics	For all the patients involved population characterisitics can be found in table 1.
Recruitment	Every patient with an active disease (as demonstrated by reddish patches or plaques) was asked to participate in the study.
Ethics oversight	Ethikkommiission der Landesärztekammer Rheinland-Pfalz

Note that full information on the approval of the study protocol must also be provided in the manuscript.

Field-specific reporting

Please select the one below that is the best fit for your research. If you are not sure, read the appropriate sections before making your selection.

☒ Life sciences ☐ Behavioural & social sciences ☐ Ecological, evolutionary & environmental sciences

For a reference copy of the document with all sections, see nature.com/documents/nr-reporting-summary-flat.pdf

Life sciences study design

All studies must disclose on these points even when the disclosure is negative.

Sample size	All patients which could be recruited during the study preiod were included. As this is an observational study we were not able to do an a priori sample size claculation
Data exclusions	No data was excluded, except for NGS runs when not sufficient material could be loaded.
Replication	We describe here a patient population. Therefore a replication of this study results can only be done if another cohort is recruited.
Randomization	This is an observational study without intervention. No randomization was applicable.
Blinding	This is an observational study without intervention. No blinding was applicable.

Reporting for specific materials, systems and methods

We require information from authors about some types of materials, experimental systems and methods used in many studies. Here, indicate whether each material, system or method listed is relevant to your study. If you are not sure if a list item applies to your research, read the appropriate section before selecting a response.

Materials & experimental systems

n/a	Involved in the study	
☒	☐	Antibodies
☒	☐	Eukaryotic cell lines
☒	☐	Palaeontology and archaeology
☒	☐	Animals and other organisms
☐	☒	Clinical data
☒	☐	Dual use research of concern
☒	☐	Plants

Methods

n/a	Involved in the study	
☒	☐	ChIP-seq
☒	☐	Flow cytometry
☒	☐	MRI-based neuroimaging

Clinical data

Policy information about clinical studies

All manuscripts should comply with the ICMJE guidelines for publication of clinical research and a completed CONSORT checklist must be included with all submissions.

Clinical trial registration	n/a
Study protocol	The study protocol can be requested from the corresponding author
Data collection	The data collection files are given in tables in the manuscript and NGS data is uploaded to the repositories as mentioned above.
Outcomes	Outcome is described in the manuscript.

Plants

Seed stocks	n/a
Novel plant genotypes	n/a
Authentication	n/a
