# Supplementary material for: The skin microbiome stratifies patients with cutaneous T cell lymphoma and determines event-free survival
Source: NPJ Biofilms Microbiomes. 2024 Aug 29;10:74. doi: 10.1038/s41522-024-00542-4 (PMC11358159; doi:10.1038/s41522-024-00542-4)
Supplement: Supplementary file 2 — Suppmenental Material 1 [file 41522_2024_542_MOESM2_ESM.pdf]

Supplementary Material 1

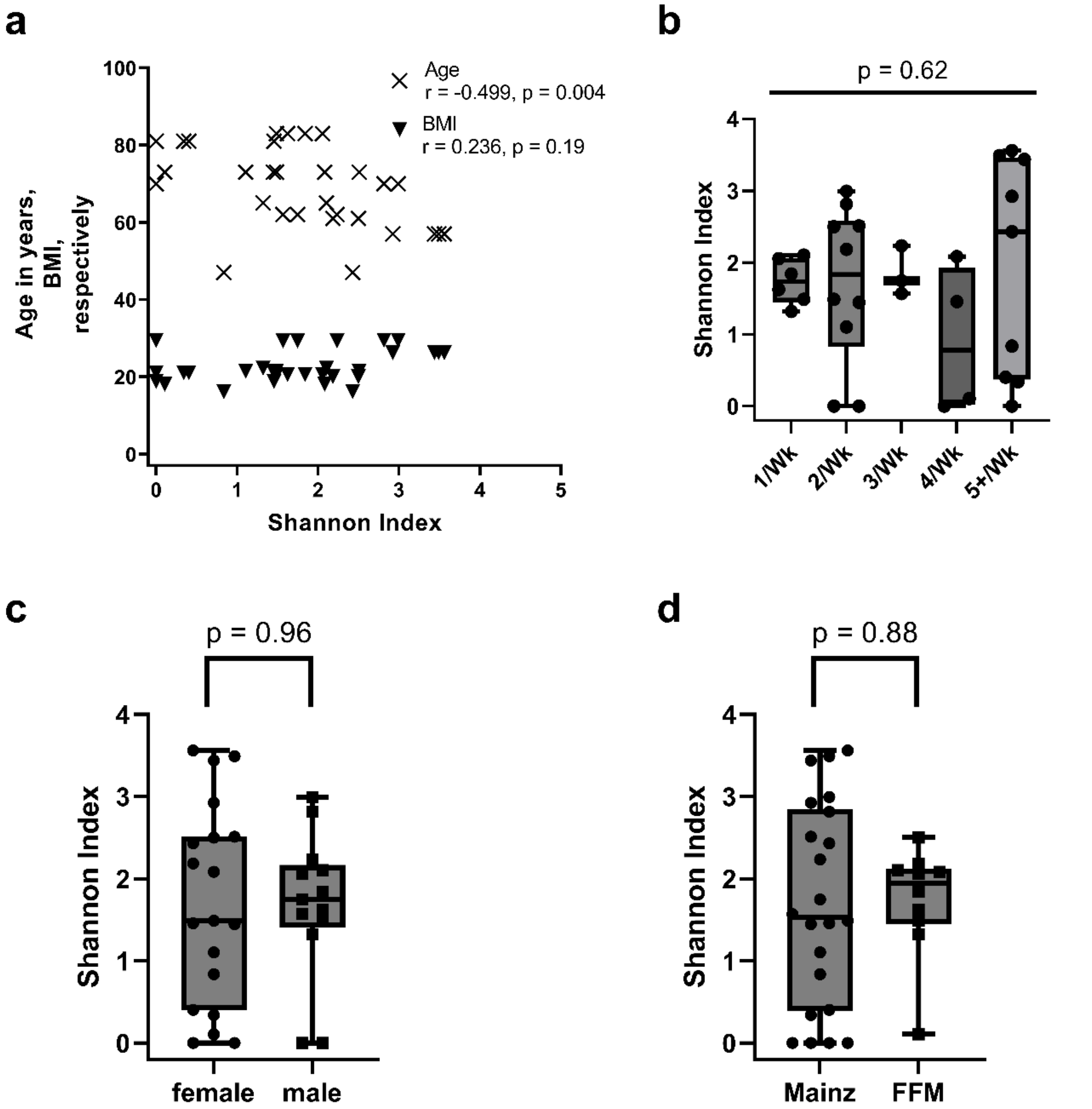

Supplementary Figure 1: Associations of α-diversity (Shannon Index) with demographic data. Reads were rarefied to common depth. a, Age in years and the BMI were correlated using spearman with the Shannon Index. **b,** Weekly Showering frequency. Wk = weekly. Kruskal-Wallis test, n = 32 **c,** Sex-related differences, Mann Whitney test, n = 32 **d,** Differences between study sites, FFM = Frankfurt am Main, Germany. Mann Whitney test, n = 32.

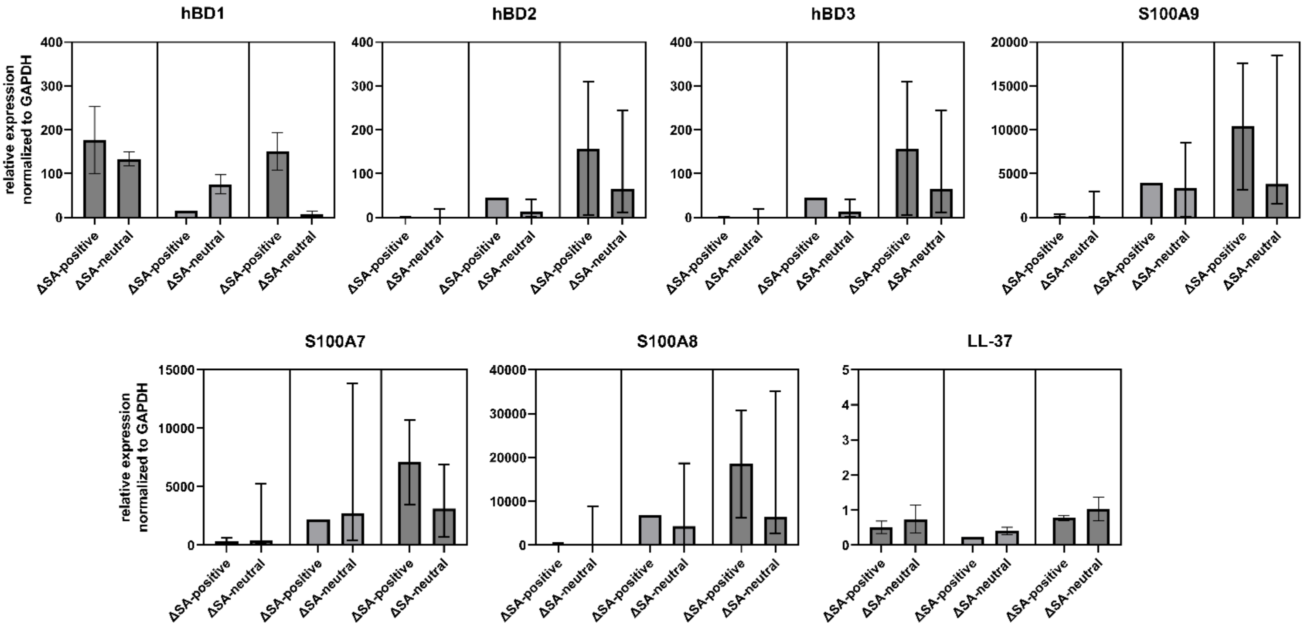

Supplementary Figure 2: Expression levels of AMPs stratified to ΔSA-positive and ΔSA-negative subgroups.

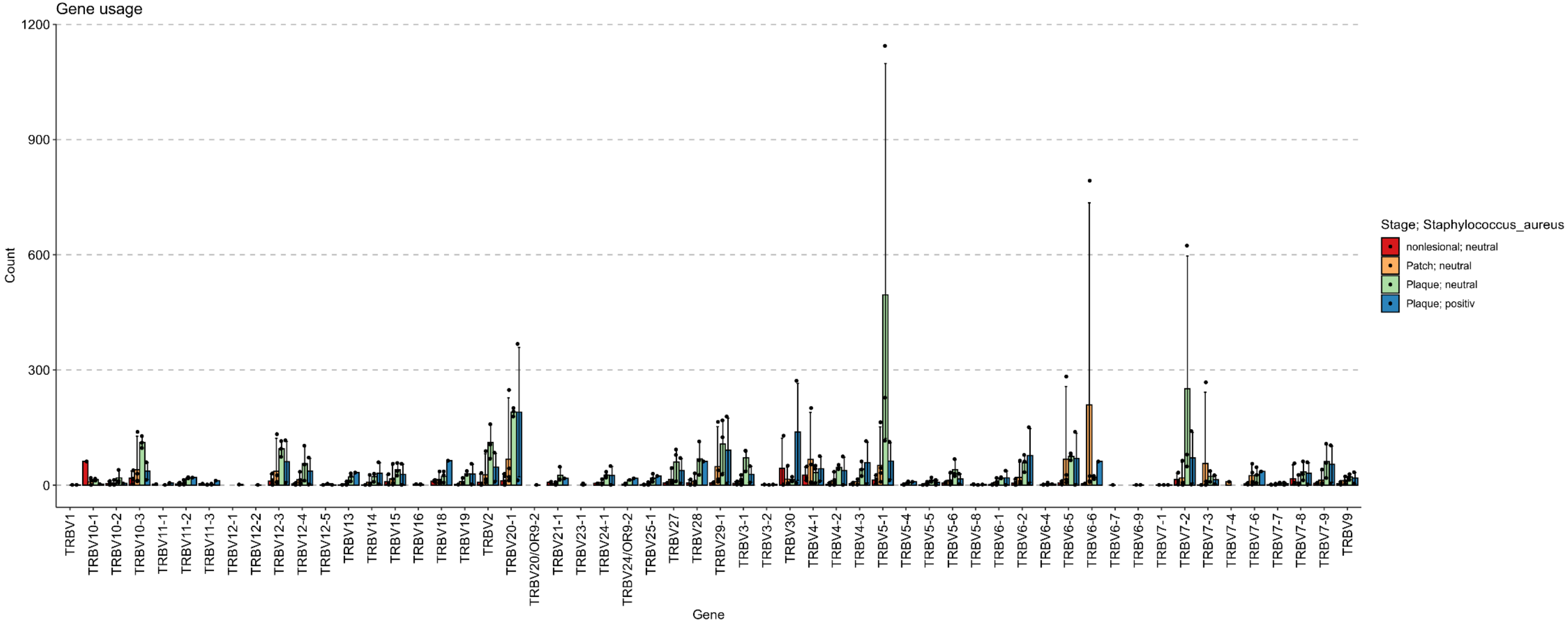

Supplementary Figure 3: TRBV Gene Usage of the TCR repertoire stratified to lesional stages and *S. aureus* status. TRBV5-1 is heavily reduced in Plaques with S. aureus. TRBV5-1 was linked to being tumour infiltrating lymphocytes by another investigation [1].

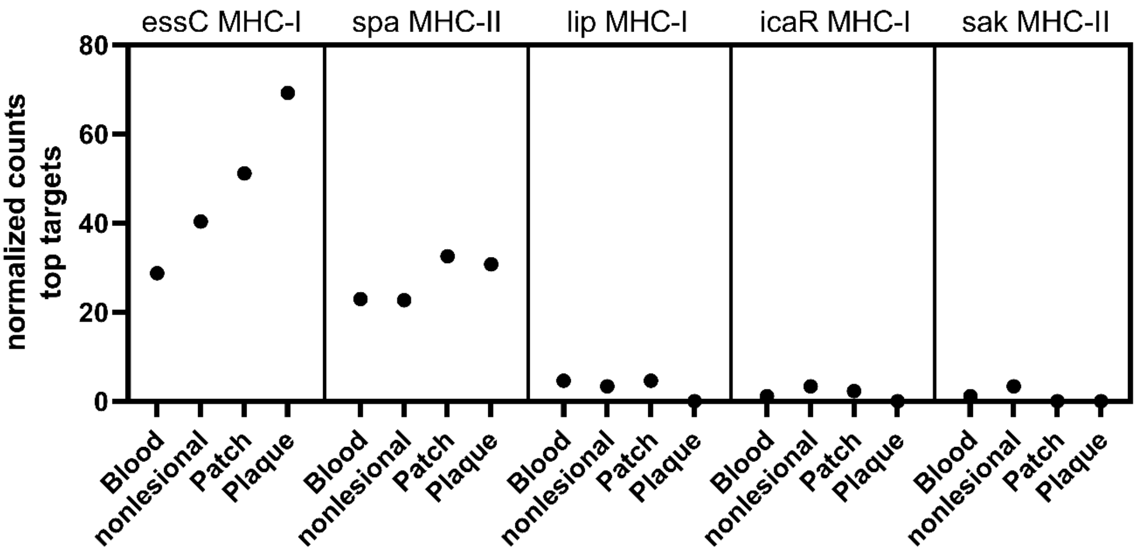

Supplementary Figure 4: Top epitope targets of T cell receptors. The epitopes that showed the highest binding score (strongest affinity) to TCRs in each sample were denoted and counted. The epitope for spa, a virulence factor enriched on Plaque of ΔSA-positive patients, is recognized by MHC-II with high affinity. spa was shown to trigger inflammation and survival via NF-κB [2,3] and CD4+ T cells are the subset of T cells that are malignant in MF [4].

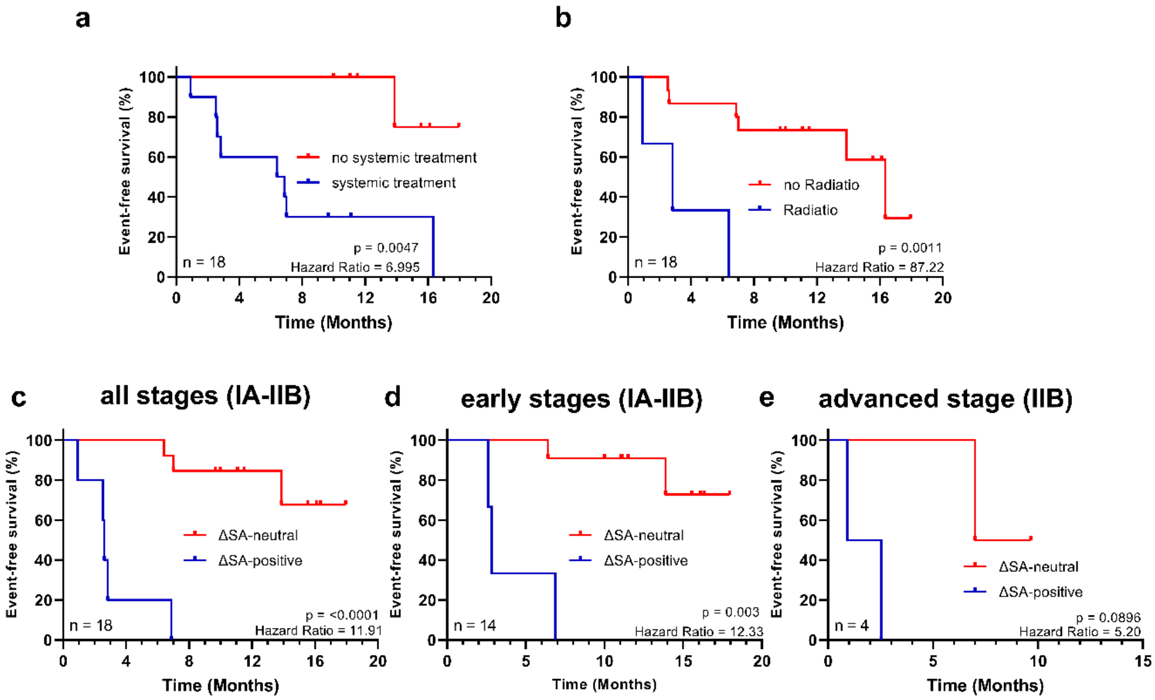

Supplementary Figure 5: Event-free survival Kaplan-Meier curves. The log-rank test was used to determine significance of differences between survival curves (1 degree of freedom). a, 95% Confidence Interval 2.801 to 39.14 **b,** 95% Confidence Interval 0.5117 to 91.12 **c,** 95% Confidence Interval 18.21 to 98.36 **d,** 95% Confidence Interval 0.74 to 206.5 **e,** 95% Confidence Interval 0.42 to 65.05.

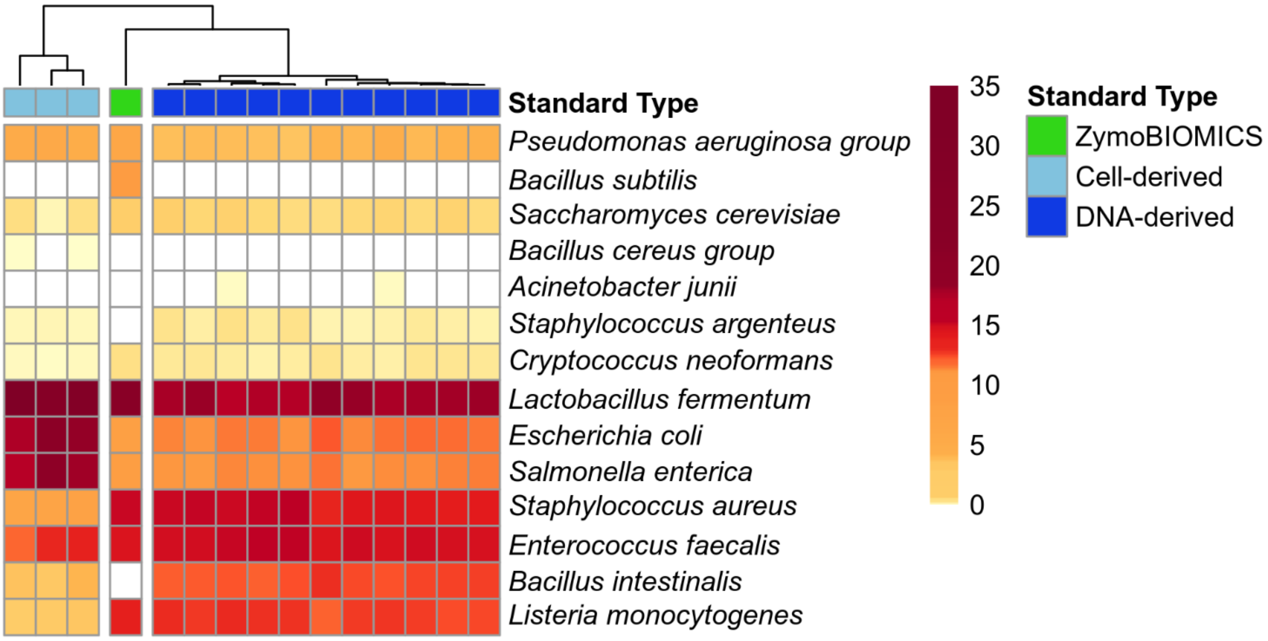

Supplementary Figure 6: High concordance of Cell- and DNA-derived positive controls with the suppliers' specifications. Control standards comprising of either a mock community of inactivated microbial cells (i.e., Cell-derived) or thereof derived microbial DNA (i.e., DNA-derived) processed with each batch of metagenomic sample DNA extraction and library preparation. The suppliers specified microbial composition of both standards is annotated as ZymoBIOMICS. Overall, both Cell-derived and DNA-derived standards showed high concordance between with the suppliers' specifications. *B. subtilis* was mistakenly classified as *B. intestinalis* and *B. cereus group* (very small read fractions in two Cell-derived control samples). A small fraction of reads belonging to *S. aureus* was mistakenly classified as *S. argenteus*. The latter organism is highly similar to *S. aureus* and was first described as a strain of this species, until *S. argenteus* was classified as individual organism [5,6]. Very small fractions of reads were mistakenly assigned to *A. junii* in two DNA-derived samples. Clustering was performed using Euclidean distance.

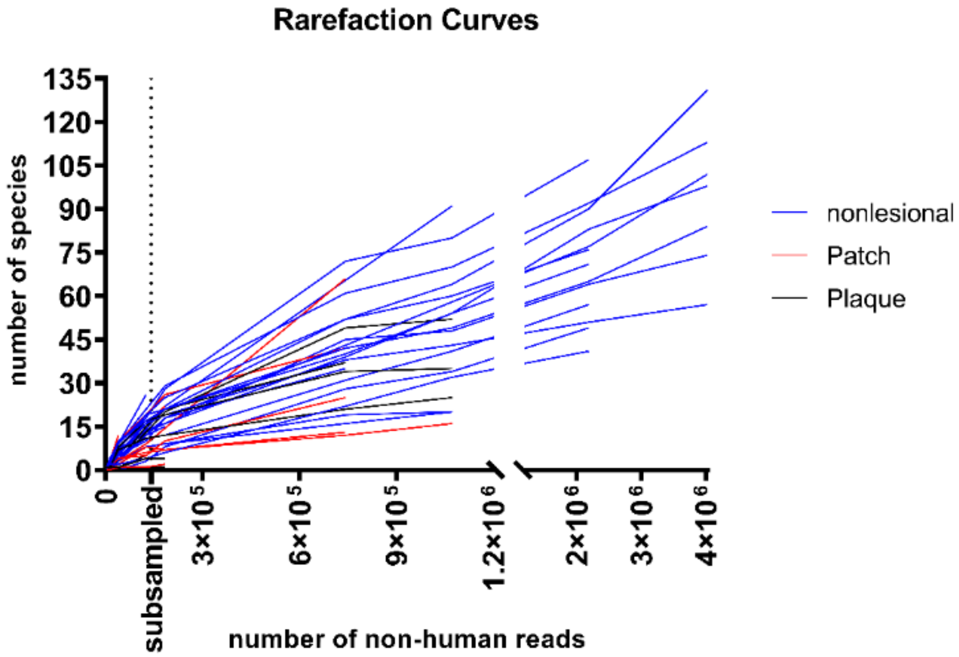

Supplementary Figure 7: Rarefaction curves. Non-human, quality-checked metagenomic reads were subsampled to increasing depths. For the analysis of diversity metrics and clustering taxonomic profiles samples were subsampled to 141,703 reads. At this point most metagenomic samples are still below the subsampling-threshold and the number of observed species are maximized.

Supplementary Table 1: Antibiotic diffusion assay. The patient derived isolates *S. aureus* MFMZ1, *S. epidermidis* MFMZ1, and *S. hominis* MFMZ1 as well as positive control *S. aureus* DSM11823 and negative control strains derived from healthy patients *S. aureus* EM01 and *S. epidermidis* MV01 were tested on resistance towards a set of commonly used antibiotics in the clinic. **a,** The antibiotics were applied using a Sensi-Disk dispenser with (1) 25 µg ampicillin, (2) 100 µg carbenicillin, (3) (23.75 µg) sulfamethoxazol + (1.25 µg) trimethoprim, (4) 15 µg erythromycin, (5) 10 µg gentamycin, and (6) 5 µg novobiocin. Measurement of halos indicating (s) sensitive, (i) intermediate, or (r) resistance effect of the antibiotics towards the tested Staphylococci strains. * indicates that the antibiotic showed an unclear categorization between i and r.

	***S. aureus* EM01**	***S. aureus* DSM 11823**	***S. aureus* MFMZ1**	***S. hominis* MFMZ1**	***S. epidermidis* MFMZ1**	***S. epidermidis* MV01**
(1) Ampicillin	24.0 mm (r)	27.1 mm (r)	0 mm (r)	34.4 mm (s)	33.4 mm (s)	36.1 mm (s)
(2) Carbenicillin	26.0 (s)	24.1 mm (i)	0 mm (r)	26.2 mm (s)	26.2 mm (s)	36.6 mm (s)
(3) Sulfamethoxazole + Trimethoprim	15.0 mm (i)	11.8 mm (i)	12.0 mm (i)	21.0 mm (s)	20.8 mm (s)	15.3 mm (s)
(4) Erythromycin	15.0 mm (i)	20.1 mm (i)	20.0 mm (i)	25.1 mm (s)	28.2 mm (s)	22.3 mm (s)
(5) Gentamycin	10.0 mm (r)	14.9 mm (s)	12.0 mm (i-r)*	14.4 mm (s)	25.9 mm (s)	15.9 mm (s)
(6) Novobiocin	24.0 mm (s)	28.5 mm (s)	29.4 mm (s)	27.0 mm (s)	38.2 mm (s)	33.1 mm (s)

Supplementary Table 2: Sequences of Primers for AMPs and quantification of Staphylococci quantification and virulence genes.

Gene	*Primers*	*Source*
GAPDH	F: 5'-TCAAGGCTGAGAACGGGAAG-3'<br>R: 5'-CGCCCCACTTGATTTTGGAG-3'	[7]
hBD1	F: 5'-TGAGAACTTCCTACCTTCTGCTGTTT-3'<br>R: 5'-GCCAAGGCCTGTGAGAAAGTTA-3'	[8]
hBD2	F: 5'-TGATGCCTCTTCCAGGTGTTT-3'<br>R: 5'-GGATGACATATGGCTCCACTCTT-3'	[9]
hBD3	F: 5'-TGAGGATCCATTATCTTCTGTTTGCT-3'<br>R: 5'-TTCTGTAATGTGTTTATGATTCCTCCA-3'	[8]
LL-37	F: 5'-TGACTTCAAGAAGGACGGGC-3'<br>R: 5'-AGGGCACACACTAGGACTCT-3'	Self-Designed
S100A7	F: 5'-CTTCCTTAGTGCCTGTGACAAAAA-3'<br>R: 5'-AAAGACAGAAACTCAGAAAAATCAATCT-3'	[10]
S100A8	F: 5'-ATGCCGTCTACAGGGATGAC-3'<br>R: 5'-ACGCCCATCTTTATCACCAG-3'	[11]
S100A9	F: 5'-CAGCTGGAACGCAACATAGA-3'<br>R: 5'-TCAGCTGCTTGTCTGCATTT-3'	[10]
spa	F: 5'-GATTTTGCGGTTTTAAGCC-3'<br>R: 5'-GAGTAGAAAGTGTTGAGGC-3'	Self-Designed

References

1. Iyer A, Hennessey D, O'Keefe S, Patterson J, Wang W, Salopek T, et al. Clonotypic heterogeneity in cutaneous T-cell lymphoma (mycosis fungoides) revealed by comprehensive whole-exome sequencing. Blood Adv [Internet]. 2019;3:1175–84. Available from: https://ashpublications.org/bloodadvances/article/3/7/1175/247276/Clonotypic-heterogeneity-in-cutaneous-Tcell

2. Gómez MI, Lee A, Reddy B, Muir A, Soong G, Pitt A, et al. Staphylococcus aureus protein A induces airway epithelial inflammatory responses by activating TNFR1. Nat Med [Internet]. 2004;10:842–8. Available from: http://www.nature.com/articles/nm1079

3. Gómez MI, O'Seaghdha M, Magargee M, Foster TJ, Prince AS. Staphylococcus aureus Protein A Activates TNFR1 Signaling through Conserved IgG Binding Domains. J Biol Chem [Internet]. 2006;281:20190–6. Available from: https://linkinghub.elsevier.com/retrieve/pii/S0021925819763339

4. Campbell JJ, Clark RA, Watanabe R, Kupper TS. Sézary syndrome and mycosis fungoides arise from distinct T-cell subsets: a biologic rationale for their distinct clinical behaviors. Blood [Internet]. 2010;116:767–71. Available from: https://ashpublications.org/blood/article/116/5/767/107723/Sézary-syndrome-and-mycosis-fungoides-arise-from

5. Holt DC, Holden MTG, Tong SYC, Castillo-Ramirez S, Clarke L, Quail MA, et al. A Very Early-Branching Staphylococcus aureus Lineage Lacking the Carotenoid Pigment Staphyloxanthin. Genome Biol Evol [Internet]. 2011;3:881–95. Available from: https://academic.oup.com/gbe/article/doi/10.1093/gbe/evr078/591113

6. Tong SYC, Schaumburg F, Ellington MJ, Corander J, Pichon B, Leendertz F, et al. Novel staphylococcal species that form part of a Staphylococcus aureus-related complex: the non-pigmented Staphylococcus argenteus sp. nov. and the non-human primate-associated Staphylococcus schweitzeri sp. nov. Int J Syst Evol Microbiol [Internet]. 2015;65:15–22. Available from: https://www.microbiologyresearch.org/content/journal/ijsem/10.1099/ijs.0.062752-0

7. Dahn ML, Dean CA, Jo DB, Coyle KM, Marcato P. Human-specific GAPDH qRT-PCR is an accurate and sensitive method of xenograft metastasis quantification. Mol Ther - Methods Clin Dev [Internet]. The Author(s); 2021;20:398–408. Available from: https://doi.org/10.1016/j.omtm.2020.12.010

8. Wolk K, Mitsui H, Witte K, Gellrich S, Gulati N, Humme D, et al. Deficient Cutaneous Antibacterial Competence in Cutaneous T-Cell Lymphomas: Role of Th2-Mediated Biased Th17 Function. Clin Cancer Res [Internet]. 2014;20:5507–16. Available from: http://clincancerres.aacrjournals.org/lookup/doi/10.1158/1078-0432.CCR-14-0707

9. Gambichler T, Skrygan M, Appelhans C, Tomi NS, Reinacher-Schick A, Altmeyer P, et al. Expression of human β-defensins in patients with mycosis fungoides. Arch Dermatol Res [Internet]. 2007;299:221–4. Available from: http://link.springer.com/10.1007/s00403-007-0749-6

10. Suga H, Sugaya M, Miyagaki T, Ohmatsu H, Kawaguchi M, Takahashi N, et al. Skin Barrier Dysfunction and Low Antimicrobial Peptide Expression in Cutaneous T-cell Lymphoma. Clin Cancer Res [Internet]. 2014;20:4339–48. Available from: http://clincancerres.aacrjournals.org/lookup/doi/10.1158/1078-0432.CCR-14-0077

11. Nakajima R, Miyagaki T, Kamijo H, Oka T, Shishido-Takahashi N, Suga H, et al. Decreased progranulin expression in Mycosis fungoides: a possible association with the high frequency of skin infections. Eur J Dermatol [Internet]. 2018;28:790–4. Available from: http://www.ncbi.nlm.nih.gov/pubmed/30530405

B. Liu, D. Zheng, S. Zhou, L. Chen, J. Yang, Nucleic Acids Res. 50 (2022).
E. Blümel *et al.*, *Oncoimmunology*. **8**, e1641387 (2019).
E. Blümel *et al.*, *Oncoimmunology*. **9**, 1751561 (2020).
F. Alonzo, V. J. Torres, *Microbiol. Mol. Biol. Rev.* **78**, 199–230 (2014).
G. Y. C. Cheung, J. S. Bae, M. Otto, *Virulence*. **12**, 547–569 (2021).
D. A. Bloes, D. Kretschmer, A. Peschel, *Nat. Rev. Microbiol.* **13**, 95–104 (2015).

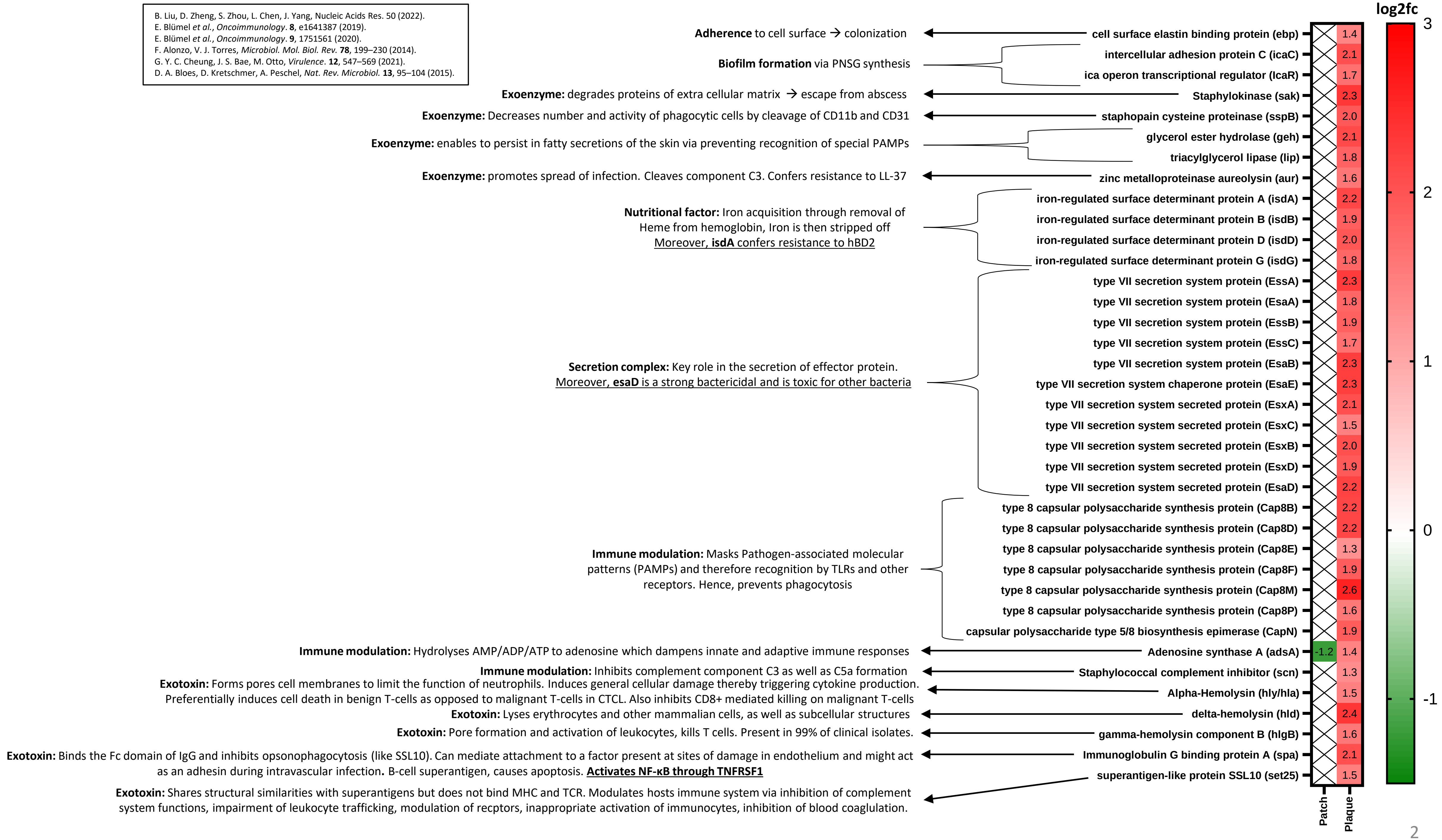

Supplementary Table 3: Differential Abundances Analysis of Microbial Species

feature	value	coef	fc	log2fc	stderr	N	N.not.zero	pval	qval
Malassezia_restricta	Plaque	-5,49759065	0,00409663	-7,93134677	0,96641785	65	43	9,57E-07	8,42E-05
Corynebacterium_matruchotii	Patch	-3,88813895	0,02048343	-5,60939878	0,75238701	65	19	5,70E-06	0,00025086
Cutibacterium_acnes	Plaque	-4,70187664	0,00907822	-6,78337412	0,96957971	65	58	1,64E-05	0,00048228
Finegoldia_magna	Plaque	-5,85585726	0,00286308	-8,44821622	1,26111871	65	44	2,56E-05	0,00056297
Micrococcus_luteus	Plaque	-4,53207265	0,01075835	-6,53839873	0,9868459	65	39	3,87E-05	0,00056718
Staphylococcus_hominis	Patch	-3,94654105	0,01932142	-5,6936552	0,85499377	65	38	3,43E-05	0,00056718
Rothia_dentocariosa	Patch	-4,94328864	0,00713111	-7,13165801	1,09690569	65	25	5,11E-05	0,00062085
Cutibacterium_granulosum	Plaque	-3,81563192	0,02202379	-5,50479324	0,86285818	65	37	6,44E-05	0,00062085
Peptoniphilus_harei	Plaque	-3,82016496	0,02192418	-5,51133305	0,87366953	65	29	7,06E-05	0,00062085
Acinetobacter_johnsonii	Patch	-4,35588335	0,0128311	-6,2842113	0,983059	65	23	5,89E-05	0,00062085

Staphylococcus_capitis	Patch	-4,04559864	0,01749923	-5,83656509	0,94172039	65	42	9,53E-05	0,00070631
Streptococcus_mitis	Patch	-3,17925775	0,04161653	-4,58669938	0,73932587	65	26	9,63E-05	0,00070631
Staphylococcus_epidermidis	Patch	-2,74912933	0,06398355	-3,96615525	0,64964189	65	52	0,00010746	0,00072745
Corynebacterium_amycolatum	Patch	-3,29483564	0,03707414	-4,75344303	0,77704325	65	23	0,00011939	0,00075047
Cutibacterium_granulosum	Patch	-3,17089086	0,0419662	-4,57462851	0,76885349	65	37	0,0001678	0,00086859
Staphylococcus_hominis	Plaque	-3,93660708	0,01951431	-5,67932351	0,95776374	65	38	0,00016682	0,00086859
Paracoccus_yeei	Plaque	-4,48238915	0,01130637	-6,4667206	1,08616949	65	24	0,00015464	0,00086859
Paracoccus_yeei	Patch	-3,92939304	0,0196556	-5,66891585	0,96834152	65	24	0,00019545	0,00095552
Malassezia_restricta	Patch	-3,46911874	0,03114446	-5,0048804	0,86147204	65	43	0,00022318	0,00103368
Staphylococcus_capitis	Plaque	-4,1502077	0,01576114	-5,98748406	1,05556515	65	42	0,00029093	0,00128007
Moraxella_osloensis	Plaque	-4,2330349	0,01450829	-6,10697846	1,11388662	65	44	0,00044957	0,00188392

Corynebacterium_pseudogenitalium	Plaque	-3,4577321	0,03150112	-4,98845295	0,92115016	65	38	0,00050582	0,00202328
Acinetobacter_johnsonii	Plaque	-4,00417735	0,01823929	-5,77680681	1,09974909	65	23	0,00068139	0,00260705
Rothia_dentocariosa	Plaque	-4,26485015	0,01405397	-6,15287817	1,23079929	65	25	0,00121146	0,00444202
Streptococcus_oralis	Patch	-2,60054086	0,07423342	-3,7517874	0,76045037	65	23	0,00133489	0,00469882
Staphylococcus_epidermidis	Plaque	-2,44323869	0,08687902	-3,52484834	0,72683001	65	52	0,00153403	0,00519212
Rothia_mucilaginosa	Patch	-2,79824457	0,0609169	-4,03701356	0,83764725	65	18	0,00176731	0,00576011
Kocuria_rhizophila	Patch	-2,11589907	0,12052488	-3,0525971	0,6543407	65	23	0,00239305	0,00745729
Anaerococcus_nagyae	Plaque	-2,49947481	0,08212812	-3,60597991	0,78032537	65	23	0,00250169	0,00745729
Anaerococcus_octavius	Plaque	-2,77957629	0,0620648	-4,01008093	0,8705023	65	25	0,00254226	0,00745729
Corynebacterium_afermentans	Plaque	-2,69099975	0,06781311	-3,88229199	0,85506832	65	27	0,00281074	0,00773277
Prevotella_timonensis	Plaque	-3,97613211	0,01875805	-5,73634608	1,2566805	65	24	0,00281192	0,00773277

Peptoniphilus_coxii	Plaque	-2,72926724	0,0652671	-3,93750032	0,86795198	65	19	0,00294075	0,00784199
Dermabacter_hominis	Patch	-2,34655636	0,09569815	-3,38536522	0,76926075	65	17	0,00381004	0,00986127
Peptoniphilus_lacrimalis	Plaque	-3,0257822	0,04851985	-4,36528097	1,00517083	65	20	0,0041929	0,01054214
Enhydrobacter_aerosaccus	Plaque	-5,06642331	0,00630493	-7,30930378	1,75291455	65	39	0,00598489	0,01462974
Prevotella_timonensis	Patch	-3,21261482	0,04025123	-4,63482347	1,12061248	65	24	0,00636736	0,015144
Corynebacterium_afermentans	Patch	-2,14749803	0,11677596	-3,09818476	0,76504052	65	27	0,00722161	0,01672372
Kocuria_rhizophila	Plaque	-2,07079863	0,12608505	-2,98753091	0,73602172	65	23	0,00742502	0,01675389
Cutibacterium_avidum	Plaque	-2,80328879	0,0606104	-4,04429084	1,01529807	65	19	0,00815313	0,01793689
Acinetobacter_lwoffii	Patch	-1,48036771	0,227554	-2,13571916	0,54827891	65	20	0,00990759	0,02126508
Staphylococcus_haemolyticus	Patch	-1,3856427	0,25016297	-1,99905985	0,51386847	65	21	0,01022423	0,02142221
Corynebacterium_tuberculostearicum	Plaque	-1,9010008	0,14941901	-2,74256443	0,71473506	65	21	0,01078414	0,02156828

Peptoniphilus_lacrimalis	Patch	-2,3925063	0,09140032	-3,45165697	0,8990547	65	20	0,01070724	0,02156828
Staphylococcus_aureus	Plaque	2,68680517	14,6846858	3,8762405	1,02214389	65	30	0,01193997	0,02334927
Veillonella_parvula	Plaque	-1,43572699	0,23794232	-2,07131621	0,55730444	65	20	0,01305539	0,02497553
Veillonella_parvula	Patch	-1,27349239	0,27985256	-1,83726116	0,4997859	65	20	0,0141245	0,02589491
Finegoldia_magna	Patch	-2,8853679	0,05583424	-4,16270596	1,13001421	65	44	0,01388915	0,02589491
Corynebacterium_matruchotii	Plaque	-2,13958039	0,11770422	-3,08676202	0,84442201	65	19	0,01491432	0,02678491
Peptoniphilus_harei	Patch	-1,96580676	0,14004286	-2,83605966	0,77902133	65	29	0,01523543	0,02681435
Peptoniphilus_coxii	Patch	-1,91676826	0,14708152	-2,76531206	0,77363977	65	19	0,01709651	0,02949987
Malassezia_globosa	Patch	-2,21282267	0,10939144	-3,1924283	0,89959872	65	22	0,01801626	0,03048906
Anaerococcus_prevotii	Plaque	-2,62930232	0,07212877	-3,79328142	1,09127044	65	18	0,01992336	0,03308029
Rothia_mucilaginosa	Plaque	-2,25663392	0,10470233	-3,25563457	0,93928573	65	18	0,02070741	0,03344629

Acinetobacter_lwoffii	Plaque	-1,47683111	0,22836019	-2,13061691	0,61592797	65	20	0,02090393	0,03344629
Micrococcus_aloeverae	Plaque	-2,34709742	0,09564638	-3,38614581	0,99919779	65	19	0,02315193	0,03588135
Streptococcus_mitis	Plaque	-1,94937507	0,14236301	-2,81235375	0,82887877	65	26	0,02324133	0,03588135
Propionibacterium_namnetense	Plaque	-0,93642585	0,3920265	-1,35097693	0,40084803	65	21	0,0244004	0,0370213
Staphylococcus_pettenkoferi	Patch	-1,0511062	0,34955086	-1,51642571	0,46108369	65	18	0,0272256	0,04060767
Actinomyces_oris	Patch	-2,61781181	0,07296234	-3,77670412	1,16467018	65	21	0,02969599	0,04284011
Kocuria_palustris	Patch	-1,82337188	0,16148034	-2,63056957	0,81112426	65	19	0,02962186	0,04284011
Streptococcus_oralis	Plaque	-1,86828214	0,15438865	-2,69536137	0,85172005	65	23	0,03332576	0,04730107
Malassezia_globosa	Plaque	-2,20347962	0,11041828	-3,17894913	1,00704544	65	22	0,03402592	0,04752827
Anaerococcus_octavius	Patch	-1,66995683	0,18825519	-2,40923844	0,77665231	65	25	0,03693405	0,05078432
Corynebacterium_tuberculostearicum	Patch	-1,347524	0,25988294	-1,94406619	0,63768257	65	21	0,04025165	0,05449454

Anaerococcus_prevotii	Patch	-2,03496965	0,13068445	-2,93584062	0,9748344	65	18	0,04236754	0,05649006
Dermabacter_hominis	Plaque	-1,72231055	0,17865288	-2,48476888	0,86198746	65	17	0,05161727	0,06779581
Faecalibacterium_prausnitzii	Plaque	-1,678031	0,18674131	-2,420887	0,8471575	65	18	0,05300957	0,06792803
Enhydrobacter_aerosaccus	Patch	-3,10278167	0,04492406	-4,47636773	1,56105133	65	39	0,05326175	0,06792803
Staphylococcus_aureus	Patch	-1,79088513	0,16681245	-2,58370109	0,90884534	65	30	0,05547613	0,06974142
Cutibacterium_avidum	Patch	-1,7732156	0,16978615	-2,55820935	0,90740494	65	19	0,05668403	0,07025626
Actinomyces_oris	Plaque	-2,5379307	0,07902977	-3,66146004	1,30451836	65	21	0,05802501	0,07091945
Staphylococcus_haemolyticus	Plaque	-1,08330035	0,33847659	-1,56287205	0,57792163	65	21	0,06816038	0,08216594
Corynebacterium_kroppenstedtii	Patch	-1,48418683	0,2266866	-2,14122897	0,80118934	65	28	0,07101495	0,08445021
Staphylococcus_pettenkoferi	Plaque	-0,91446118	0,40073249	-1,31928861	0,51557823	65	18	0,08246951	0,09676423
Faecalibacterium_prausnitzii	Patch	-1,3174793	0,26780952	-1,90072085	0,76059132	65	18	0,08944761	0,10357091

Propionibacterium_namnetense	Patch	-0,60847385	0,54418074	-0,8778422	0,35630347	65	21	0,09521696	0,10881938
Corynebacterium_kroppenstedtii	Plaque	-1,4970951	0,22377927	-2,15985168	0,90073816	65	28	0,10389972	0,11722019
Kocuria_palustris	Plaque	-1,40860988	0,24448291	-2,03219449	0,90929245	65	19	0,12836	0,141196
Anaerococcus_nagyae	Patch	-1,08152017	0,33907968	-1,56030378	0,6958318	65	23	0,12726176	0,141196
Micrococcus_luteus	Patch	-1,10972418	0,32964987	-1,60099357	0,8778293	65	39	0,21311585	0,23153327

Supplementary Table 4: Differential Abundances Analysis of Virulence Genes

feature	value	coef	fc	log2fc	stderr	N	N.not.0	pval	qval
(cap8M) type 8 capsular polysaccharide synthesis protein Cap8M [Capsule (VF0003) - Immune modulation (VFC0258)] [Staphylococcus aureus subsp. aureus MW2]	Plaque	1,79447686	6,01632653	2,58888287	0,56140402	65	17	0,00238604	0,07158114
(hld) delta-hemolysin [-hemolysin (VF0007) - Exotoxin (VFC0235)] [Staphylococcus aureus subsp. aureus MW2]	Plaque	1,63885211	5,14925533	2,36436381	0,59339576	65	7	0,00776988	0,10391755
(sak) Staphylokinase precursor [Staphylokinase (VF0021) - Exoenzyme (VFC0251)] [Staphylococcus aureus subsp. aureus MW2]	Plaque	1,62416901	5,07420067	2,34318058	0,43220667	65	14	0,00044645	0,05357401

(essA) type VII secretion system protein EssA, monotopic membrane protein [Type VII secretion system (VF0403) - Effector delivery system (VFC0086)] [Staphylococcus aureus subsp. aureus MW2]	Plaque	1,59685407	4,93747502	2,30377345	0,64946357	65	10	0,01731959	0,10391755
(esaE) type VII secretion system chaperone protein [Type VII secretion system (VF0403) - Effector delivery system (VFC0086)] [Staphylococcus aureus subsp. aureus MW2]	Plaque	1,571273	4,81277094	2,26686776	0,44427223	65	15	0,00093933	0,05635985
(esaB) type VII secretion system protein EsaB [Type VII secretion system (VF0403) - Effector delivery system (VFC0086)] [Staphylococcus aureus subsp. aureus MW2]	Plaque	1,56951905	4,80433698	2,26433735	0,62031179	65	12	0,01444507	0,10391755
(esaD) type VII secretion system secreted protein, a nuclease toxin EsaD [Type VII secretion system (VF0403) - Effector delivery system (VFC0086)] [Staphylococcus aureus subsp. aureus MW2]	Plaque	1,53071132	4,62146299	2,20834963	0,57653013	65	13	0,01044238	0,10391755
(cap8B) type 8 capsular polysaccharide synthesis protein Cap8B [Capsule (VF0003) - Immune modulation (VFC0258)] [Staphylococcus aureus subsp. aureus MW2]	Plaque	1,50781735	4,51686132	2,17532062	0,60849744	65	12	0,01665397	0,10391755
(cap8D) type 8 capsular polysaccharide synthesis protein Cap8D [Capsule (VF0003) - Immune modulation (VFC0258)] [Staphylococcus aureus subsp. aureus MW2]	Plaque	1,50669137	4,51177829	2,17369617	0,59512364	65	11	0,01455379	0,10391755

(isdA) iron-regulated surface determinant protein A [Isd (VF0015) - Nutritional/Metabolic factor (VFC0272)] [Staphylococcus aureus subsp. aureus str. Newman]	Plaque	1,49726282	4,46943864	2,16009364	0,56152265	65	10	0,01010552	0,10391755
(geh) glycerol ester hydrolase [Lipase (VF0012) - Exoenzyme (VFC0251)] [Staphylococcus aureus subsp. aureus MW2]	Plaque	1,47054387	4,35160122	2,12154635	0,52854085	65	8	0,00734715	0,10391755
(spa) Immunoglobulin G binding protein A precursor [SpA (VF0017) - Exotoxin (VFC0235)] [Staphylococcus aureus subsp. aureus MW2]	Plaque	1,43449496	4,19752456	2,06953877	0,65002904	65	9	0,03153937	0,14556632
(esxA) type VII secretion system secreted protein EsxA [Type VII secretion system (VF0403) - Effector delivery system (VFC0086)] [Staphylococcus aureus subsp. aureus MW2]	Plaque	1,42815607	4,17100105	2,06039368	0,49629191	65	21	0,00595724	0,10391755
(icaC) intercellular adhesion protein C, involved in polysaccharide intercellular adhesin(PIA) synthesis [Intercellular adhesion proteins (VF0014) - Biofilm (VFC0271)] [Staphylococcus aureus subsp. aureus MW2]	Plaque	1,42733293	4,16756916	2,05920614	0,57095955	65	13	0,01585216	0,10391755
(esxB) type VII secretion system secreted protein EsxB [Type VII secretion system (VF0403) - Effector delivery system (VFC0086)] [Staphylococcus aureus subsp. aureus MW2]	Plaque	1,42065402	4,13982707	2,04957051	0,43971082	65	13	0,00228099	0,07158114
(isdD) iron-regulated surface determinant protein D [Isd (VF0015) - Nutritional/Metabolic factor (VFC0272)] [Staphylococcus aureus subsp. aureus str. Newman]	Plaque	1,37213874	3,94377641	1,97957776	0,50296924	65	11	0,00881067	0,10391755

(sspB) staphopain cysteine proteinase SspB [Staphopain (VF0006) - Exoenzyme (VFC0251)] [Staphylococcus aureus subsp. aureus MW2]	Plaque	1,35391851	3,87257054	1,95329152	0,54207889	65	12	0,01575994	0,10391755
(esxD) type VII secretion system secreted protein EsxD [Type VII secretion system (VF0403) - Effector delivery system (VFC0086)] [Staphylococcus aureus subsp. aureus MW2]	Plaque	1,34144719	3,82457438	1,9352992	0,55125895	65	14	0,01873092	0,10703384
capN) capsular polysaccharide type 5/8 biosynthesis epimerase CapN [Capsule (VF0003) - Immune modulation (VFC0258)] [Staphylococcus aureus subsp. aureus MW2]	Plaque	1,32550829	3,76409814	1,91230424	0,58363711	65	19	0,02740781	0,1426702
(cap8F) type 8 capsular polysaccharide synthesis protein Cap8F [Capsule (VF0003) - Immune modulation (VFC0258)] [Staphylococcus aureus subsp. aureus MW2]	Plaque	1,31557888	3,72690779	1,89797913	0,52541653	65	13	0,01566541	0,10391755
isdB) iron-regulated surface determinant protein B, haemoglobin receptor [Isd (VF0015) - Nutritional/Metabolic factor (VFC0272)] [Staphylococcus aureus subsp. aureus str. Newman]	Plaque	1,29881047	3,66493451	1,87378742	0,52623901	65	15	0,01718483	0,10391755
(essB) type VII secretion system protein EssB, monotopic membrane protein [Type VII secretion system (VF0403) - Effector delivery system (VFC0086)] [Staphylococcus aureus subsp. aureus MW2]	Plaque	1,29315565	3,64426846	1,86562924	0,5697769	65	13	0,02762377	0,1426702
(lip) triacylglycerol lipase precursor [Lipase (VF0012) - Exoenzyme (VFC0251)] [Staphylococcus aureus subsp. aureus MW2]	Plaque	1,27337489	3,57289036	1,83709164	0,49845452	65	9	0,01388927	0,10391755

(isdG) iron-regulated surface determinant protein G [Isd (VF0015) - Nutritional/Metabolic factor (VFC0272)] [Staphylococcus aureus subsp. aureus str. Newman]	Plaque	1,2435211	3,46780247	1,79402172	0,55097313	65	12	0,02853404	0,1426702
(esaA) type VII secretion system protein EsaA [Type VII secretion system (VF0403) - Effector delivery system (VFC0086)] [Staphylococcus aureus subsp. aureus MW2]	Plaque	1,24247471	3,46417571	1,79251211	0,56655199	65	17	0,03300159	0,14667374
(essC) type VII secretion system protein EssC, FtsK/SpoIIIE family ATPase [Type VII secretion system (VF0403) - Effector delivery system (VFC0086)] [Staphylococcus aureus subsp. aureus MW2]	Plaque	1,15810679	3,18389978	1,67079493	0,55363047	65	10	0,04168571	0,16606933
(icaR) ica operon transcriptional regulator IcaR [Intercellular adhesion proteins (VF0014) - Biofilm (VFC0271)] [Staphylococcus aureus subsp. aureus MW2]	Plaque	1,15236021	3,1656557	1,66250435	0,57975442	65	17	0,05198515	0,17704151
aur) zinc metalloproteinase aureolysin [Aureolysin (VF0024) - Exoenzyme (VFC0251)] [Staphylococcus aureus subsp. aureus MW2]	Plaque	1,11031699	3,03532041	1,60184881	0,5025413	65	7	0,03135327	0,14556632
(hlgB) gamma-hemolysin component B [-hemolysin (VF0011) - Exotoxin (VFC0235)] [Staphylococcus aureus subsp. aureus MW2]	Plaque	1,09618961	2,99274077	1,58146732	0,53335407	65	8	0,04465572	0,16606933
(cap8P) type 8 capsular polysaccharide synthesis protein Cap8P [Capsule (VF0003) - Immune modulation (VFC0258)] [Staphylococcus aureus subsp. aureus MW2]	Plaque	1,09553203	2,99077343	1,58051862	0,58247511	65	14	0,0659863	0,19822864

(set25) superantigen-like protein SSL10 [SSLs (VF0990) - Exotoxin (VFC0235)] [Staphylococcus aureus subsp. aureus MW2]	Plaque	1,03892936	2,82618956	1,49885823	0,54956235	65	13	0,06444136	0,19822864
(esxC) type VII secretion system secreted protein EsxC [Type VII secretion system (VF0403) - Effector delivery system (VFC0086)] [Staphylococcus aureus subsp. aureus MW2]	Plaque	1,03131103	2,80474054	1,48786731	0,49975523	65	16	0,04422772	0,16606933
(hly/hla) Alpha-Hemolysin precursor [-hemolysin (VF0001) - Exotoxin (VFC0235)] [Staphylococcus aureus subsp. aureus MW2]	Plaque	1,03114068	2,80426279	1,48762155	0,49657481	65	11	0,04270114	0,16606933
(adsA) Adenosine synthase A [AdsA (VF0422) - Immune modulation (VFC0258)] [Staphylococcus aureus subsp. aureus MW2]	Plaque	1,00479985	2,73136055	1,44961977	0,48931096	65	11	0,04566907	0,16606933
(ebp) cell surface elastin binding protein [EbpS (VF0008) - Adherence (VFC0001)] [Staphylococcus aureus subsp. aureus MW2]	Plaque	0,99864452	2,71459974	1,44073949	0,51599598	65	16	0,05838319	0,18935088
scn) complement inhibitor SCIN [SCIN (VF0425) - Immune modulation (VFC0258)] [Staphylococcus aureus subsp. aureus MW2]	Plaque	0,92725376	2,52755837	1,33774441	0,49284981	65	9	0,06607621	0,19822864
(cap8E) type 8 capsular polysaccharide synthesis protein Cap8E [Capsule (VF0003) - Immune modulation (VFC0258)] [Staphylococcus aureus subsp. aureus MW2]	Plaque	0,89285938	2,44210258	1,2881238	0,44915379	65	11	0,05208613	0,17704151
(lpxC) UDP-3-O-acyl-N-acetylglucosamine deacetylase [LPS (VF0466) - Immune modulation (VFC0258)] [Acinetobacter baumannii ACICU]	Plaque	-0,34595691	0,70754298	-0,49911032	0,16390827	65	8	0,04042482	0,16606933
(pilH) twitching motility protein PilH [Type IV pili (VF0082) - Adherence (VFC0001)] [Pseudomonas aeruginosa PAO1]	Patch	-0,63699687	0,52887833	-0,91899222	0,32202245	65	19	0,05311245	0,17704151

(pilG) twitching motility response regulator PilG [TFP (VF1334) - Adherence (VFC0001)] [Acinetobacter baumannii ACICU]	Plaque	-0,79383656	0,45210693	-1,14526407	0,31467571	65	8	0,01511838	0,10391755
(adsA) Adenosine synthase A [AdsA (VF0422) - Immune modulation (VFC0258)] [Staphylococcus aureus subsp. aureus MW2]	Patch	-0,81071167	0,44454159	-1,16960971	0,44149748	65	11	0,07289897	0,21336284
